# Field detection of multiple RNA viruses/viroids in apple using a CRISPR/Cas12a‐based visual assay

**DOI:** 10.1111/pbi.13474

**Published:** 2020-09-17

**Authors:** Jian Jiao, Kangkang Kong, Jinmeng Han, Shangwei Song, Tuanhui Bai, Chunhui Song, Miaomiao Wang, Zhenli Yan, Hengtao Zhang, Ruiping Zhang, Jiancan Feng, Xianbo Zheng

**Affiliations:** ^1^ College of Horticulture Henan Agricultural University Zhengzhou China; ^2^ Zhengzhou Fruit Research Institute Chinese Academy of Agricultural Sciences Zhengzhou Henan China

**Keywords:** apple viral diseases, LbCas12a, RT‐RPA, visualized detection, in‐field application

## Abstract

Co‐infection of apple trees with several viruses/viroids is common and decreases fruit yield and quality. Accurate and rapid detection of these viral pathogens helps to reduce losses and prevent virus spread. Current molecular detection assays used for apple viruses require specialized and expensive equipment. Here, we optimized a CRISPR/Cas12a‐based nucleic acid detection platform for the diagnosis of the most prevalent RNA viruses/viroid in apple, namely *Apple necrotic mosaic virus* (ApNMV), *Apple stem pitting virus* (ASPV), *Apple stem grooving virus* (ASGV), *Apple chlorotic leaf spot virus* (ACLSV) and *Apple scar skin viroid* (ASSVd). We detected each RNA virus/viroid directly from crude leaf extracts after simultaneous multiplex reverse transcription‐recombinase polymerase amplification (RT‐RPA) with high specificity. Positive results can be distinguished by the naked eye via oligonucleotide‐conjugated gold nanoparticles. The CRISPR/Cas12a‐RT‐RPA platform exhibited comparable sensitivity to RT‐qPCR, with limits of detection reaching 250 viral copies per reaction for ASPV and ASGV and 2500 copies for the others. However, this protocol was faster and simpler, requiring an hour or less from leaf harvest. Field tests showed 100% agreement with RT‐PCR detection for 52 samples. This novel Cas12a‐based method is ideal for rapid and reliable detection of apple viruses in the orchard without the need to send samples to a specialized laboratory.

## Introduction

Apple (*Malus* × *domestica* Borkh.) is an economically important fruit crop and is widely grown around the world. China now produces nearly half of the apples worldwide. Currently, quite a few orchards are suffering from viral pathogens, including apple stem pitting virus (ASPV), apple stem grooving virus (ASGV), apple chlorotic leaf spot virus (ACLSV), a novel apple necrotic mosaic virus (ApNMV) and apple scar skin viroid (ASSVd) (Ji *et al*., 2013; Noda *et al*., 2017; Puchta *et al*., 1990). Apple trees are generally propagated vegetatively through grafting, cuttings or layering, which facilitates viral transmission and accumulation. Co‐infections with viral particles occur frequently and can cause complex disease symptoms, resulting in extensive losses (Brakta *et al*., 2013). On most commercially grown apple cultivars, the latent viruses ACLSV, ASGV and ASPV are always symptomless (Chen *et al*., 2014; Ioanna *et al*., 2017), but susceptible cultivars do react with a variety of symptoms, such as malformation of young leaves, stem grooving, deformation on top‐grafting unions or leaf epinasty (Brunt *et al*., 1996). ApNMV and ASSVd cause symptoms such as leaf mosaic and dappled fruit, respectively, in apple varieties (Walia *et al*., 2014; Xing *et al*., 2018).

The main strategies for controlling apple viruses/viroids rely on producing virus‐free plant material via virus eradication (Hu *et al*., 2019); hence, on‐site detection in nurseries is indispensable and should be rapid, sensitive and user‐friendly. For established orchards, timely virus detection and eradication can prevent spread (Ji *et al*., 2013). Thus far, several diagnostic methods have been developed, including indexing on indicator plants, serological techniques (Jeong *et al*., 2014), molecular methods based on reverse transcription polymerase chain reaction (RT‐PCR) (Komorowska *et al*., 2010; Menzel *et al*., 2002), isothermal methods such as recombinase polymerase amplification (RPA) (Kim *et al*., 2018) and loop‐mediated isothermal amplification (Zhao *et al*., 2014). Existing diagnostic assays, however, have a variety of limitations, such as the time and expertise required, the reliance on expensive equipment (Schrader *et al*., 2012), inhibition by crude plant extracts and nonspecific amplification (Lau *et al*., 2017; Ward and Harper, 2012), that greatly restrict application outside the laboratory. Therefore, there is an urgent need to develop on‐site testing methods for rapidly and conveniently detecting apple viruses.

Recent advances have shown that CRISPR‐associated (Cas) endoribonuclease systems, such as Cas12a, Cas13a and Cas14, possess features that can be applicable for nucleic acid detection (Chen *et al*., 2018; Gootenberg *et al*., 2017; Harrington *et al*., 2018). Cas12a harbours a DNA‐activated general DNase activity, that can indiscriminately cleave nonspecific ssDNA molecules when the Cas12a/crRNA/target DNA ternary complex is formed (Chen *et al*., 2018; Li *et al*., 2018a). Cas13a has a similar nonspecific collateral cleavage activity, where RNase activities can be activated upon the recognition of specific RNA targets (Gootenberg *et al*., 2017). Researchers have combined these enzymes with a quenched fluorescent reporter and a sequence amplification step (PCR or RPA) to develop nucleic acid detection platforms called SHERLOCK (Gootenberg *et al*., 2017), DETECTR (Chen *et al*., 2018) and HOLMES (Li *et al*., 2018b), which provide attomolar sensitivity and specificity for detection of pathogenic viruses (Chaijarasphong *et al*., 2019; Myhrvold *et al*., 2018), a microorganism (Zhang *et al*., 2020a) and transgenes (Zhang *et al*., 2020b). While the newly developed detection technologies are appealing to diagnosticians and epidemiologists, they still require expensive quenched fluorescent reporters and equipment. The use of lateral flow assays lends a detection platform a visual readout that requires no additional instrumentation (Abudayyeh et al., 2019; Myhrvold *et al*., 2018; Zhang *et al*., 2020a), but does requires expensive double‐labelled probes and commercial lateral flow strips.

CRISPR‐based nucleic acid detection shows great promise in molecular diagnosis of viral diseases, but it has yet to be harnessed for detection of plant RNA viruses or viroids. Moreover, such a detection system should be paired with methods enabling direct detection from crude extracts with a visual readout. Here, we developed a specific and sensitive method for the simultaneous detection of ASPV, ASGV, ACLSV, ApNMV and ASSVd using a CRISPR/Cas12a system. The developed assay has high specificity, sensitivity and reproducibility, comparable to conventional RT‐PCR and RT‐qPCR. To the best of our knowledge, this is the first Cas12a‐based visual detection platform that is capable of simultaneously diagnosing five different RNA viruses/viroids of apple in the field, not requiring technical expertise or non‐portable equipment. Our work contributes to a better understanding of how to design, develop and use CRISPR/Cas12a assays for simultaneous detection of plant RNA viruses. This technique has the potential to be adapted to any plant species and to be used to rapidly detect nucleotide sequence.

## Results

### Optimized conditions for visual detection of nucleic acids based on Cas12a activity and AuNP linkage

**Figure 1:**
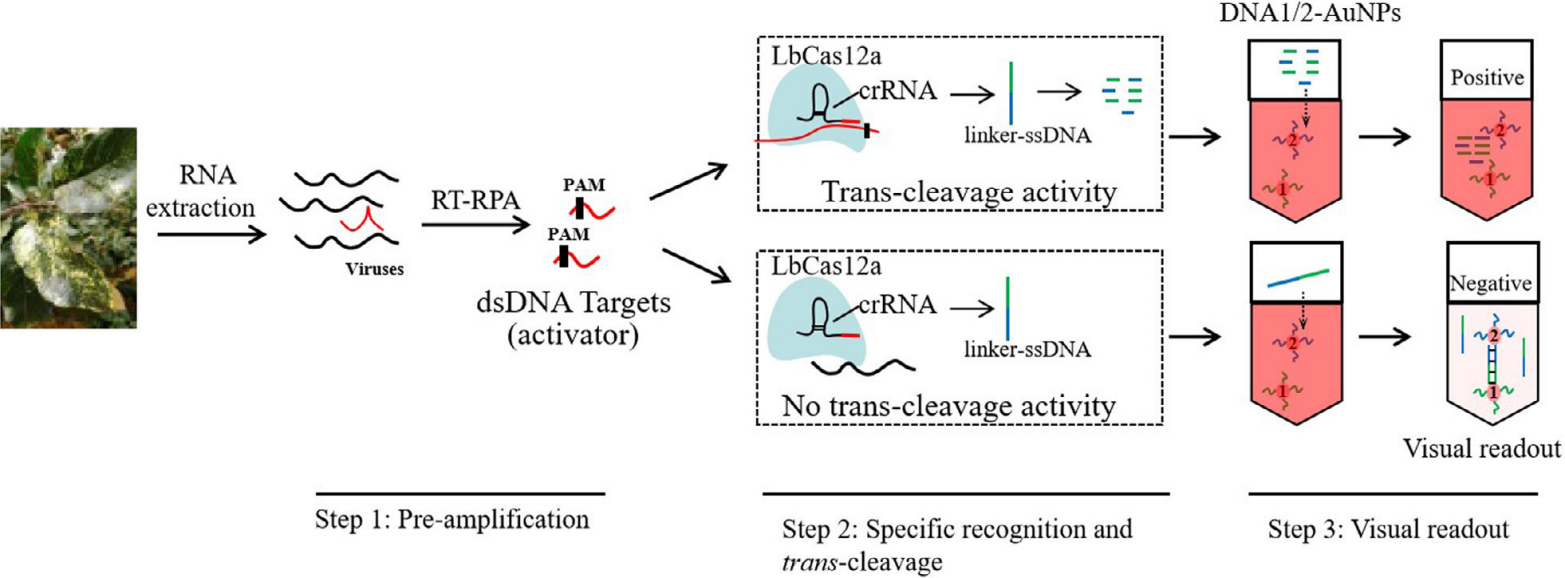
Schematic of the method for visual field detection of virus/viroid RNA in apple using RT‐RPA/Cas12a/AuNPs assay. The virus/viroid target is shown in red. The unique genomic regions of each RNA virus/viroid were amplified by RT‐RPA and converted to dsDNA. The amplification product was added into a Cas12a system with a virus‐specific crRNA, allowing the indiscriminative ssDNase activity of Cas12a to degrade a nonspecific linker‐ssDNA. The complementary linker‐ssDNA (green/blue line) is designed as a hybridization template for the DNA1/2‐AuNP pair. The reaction from step 2 is mixed with an equal volume of the DNA1/2‐AuNPs. In step 3, degradation of linker‐ssDNA by Cas12a prevents aggregation of DNA1/2‐AuNPs, keeping the solution red and representing a positive result. Conversely, when there is no viral target, the linker‐ssDNA remains intact and cross‐links the two DNA‐AuNPs, resulting in a colourless solution, representing a negative result. Red positives and clear negatives are easily distinguished by the naked eye. RT‐RPA, reverse transcription‐recombinase polymerase amplification; PAM, a 5’TTTN protospacer‐adjacent motif sequence; dsDNA double‐stranded DNA; and DNA‐AuNPs, DNA‐conjugated gold nanoparticles. [Colour figure can be viewed at wileyonlinelibrary.com]

**Figure 2:**
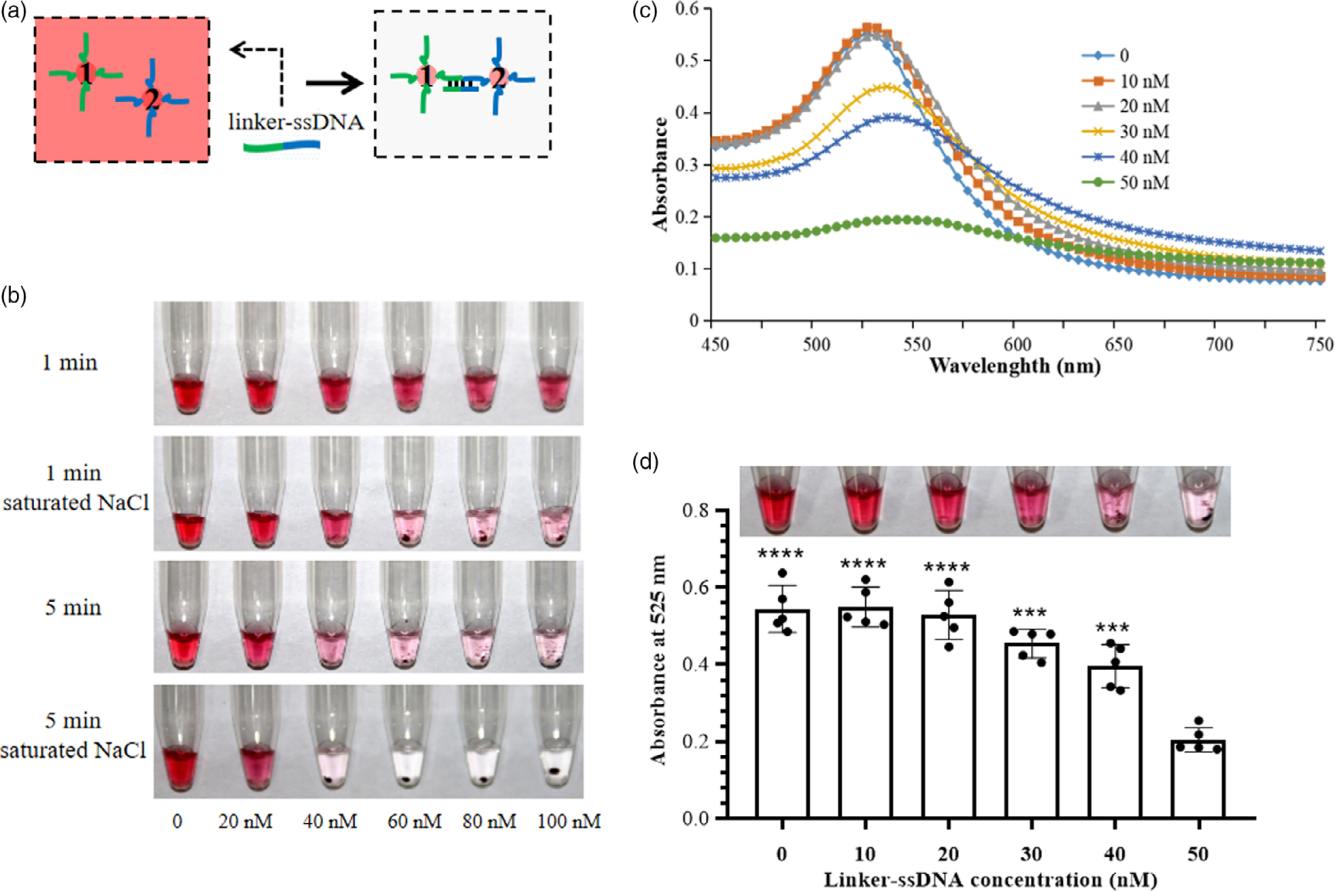
The optimal concentration of linker‐ssDNA for visual detection. (a) Schematic illustration of colour transition. The linker‐ssDNA contains one portion that is complementary to DNA1 (green) and one portion that is complementary to DNA2. (b) NaCl accelerates the irreversible aggregation of the gold nanoparticles leading to a sharp change from red to colourless. (c) Absorbance spectra of the DNA1/2‐AuNP mixture with 5 µL of saturated NaCl solution and varying linker‐ssDNA concentrations from 0 to 50 nm. (d) Determination of optimized linker‐ssDNA concentration by both direct observation and absorption measurement at 525 nm. The bar graph represents the absorbance (A525) signals. Data points represent replicates from five independent experiments, and the error bars indicate the mean ± SD. Asterisks indicate significant increases of absorption compared to the absorption at 50 nm linker‐ssDNA with two‐tailed Student’s *t*‐test (***P < 0.001, ****P < 0.0001). [Colour figure can be viewed at wileyonlinelibrary.com]

As shown in Figure 1, this CRISPR/Cas12a‐based nucleic acid detection platform integrates (i) pre‐amplification of virus/viroid sequences by RT‐RPA, (ii) sequence‐specific recognition and trans‐cleavage by LbCas12a/crRNA, and (iii) visual readout of oligonucleotide‐conjugated gold nanoparticles. We first determined the dose of linker‐ssDNA that would be sufficient to cause full cross‐linking of the DNA1/2‐AuNP mixture in step 3 (and thus a complete removal of red colour, as shown in Figure 2a) by both direct visual observation and measuring the absorbance spectrum. Since the cross‐linked AuNPs are not stable in a high ionic‐strength environment, 5 µL of a saturated NaCl solution was added to each reaction to accelerate the irreversible aggregation and colour transition. It took just five minutes for the red colour to fade when the linker‐ssDNA concentration was greater than 40 nm (Figure 2b). The maximum absorption peak moves to longer wavelengths when the linker‐ssDNA concentration varies from 0 to 50 nm, but the absorption value decreased sharply at 50 nm linker‐ssDNA (Figure 2c). Consistent with the changes in absorbance, the colour transition was distinctly observed at 50 nm linker‐ssDNA (Figure 2d). In the actual detection protocol, the linker‐ssDNA is already present in the LbCas12a/crRNA system. Therefore, in subsequent assays, the linker‐ssDNA concentration in the 2x LbCas12a/crRNA system solution was doubled to 100 nm.

**Figure 3:**
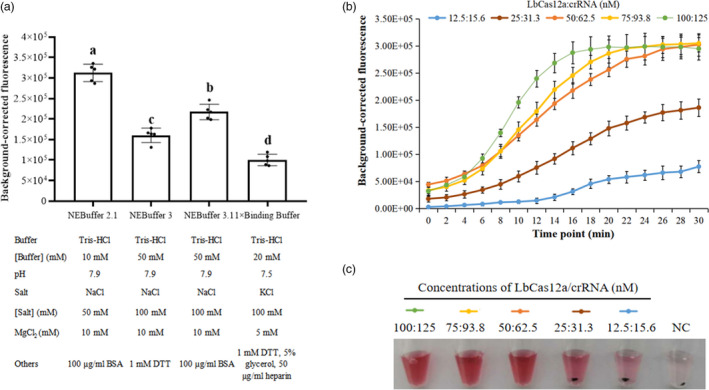
The optimization of buffer types, complex concentrations and incubation time for the trans‐cleavage activity of the LbCas12a/crRNA system. (a) Four buffers were evaluated for their effect on LbCas12a trans‐cleavage activity after 30 min of incubation with 50 nm LbCas12a and 62.5 nm crRNA (50:62.5). (b) Time courses of fluorescence detection in NEBuffer 2.1 with different concentrations of LbCas12a and crRNA with 100 nm FQ‐labelled linker‐ssDNA. (c) Visual analysis of linker‐ssDNA degradation after 30 min of incubation with different concentrations of LbCas12a/crRNA complexes as in (b). NC, negative control. Data points represent replicates from five independent experiments, and the error bars indicate the mean ± SD. Bars carrying different letters indicate means significantly different at *P* < 0.05 (one‐way ANOVA). [Colour figure can be viewed at wileyonlinelibrary.com]

After optimizing the concentration of the linker‐ssDNA, it was necessary to optimize the buffer types, the concentration of the LbCas12a/crRNA complex and the incubation time for the trans‐cleavage activity of LbCas12a. This was done in assays using FQ‐labelled linker‐ssDNA fixed at 100 nm. Preliminary fluorescence detection experiments using a 40‐bp dsDNA target (target‐40) and its crRNA revealed that the optimal buffer was the commercially available NEBuffer 2.1 (Figure 3a). The trans‐cleavage activity of LbCas12a was remarkably strengthened within 30 min when the complex was formed with 100 nm LbCas12a and 125 nm crRNA (Figure 3b).

After fluorescence detection, the reaction fluid was immediately mixed with an equal volume of the DNA1/2‐AuNP solution. Similar to what was observed in the fluorescence analysis, when the LbCas12a:crRNA complex ratio was 50:62.5 nm or higher, the linker‐ssDNA degraded completely in the LbCas12a/crRNA system, resulting in un‐cross‐linked DNA1/2‐AuNP remaining in solution, imparting the red colour to the mixture (Figure 3c).

### Design of target site for virus/viroid‐specific crRNAs

At the optimum reaction conditions, we investigated the rate and specificity of the LbCas12a/crRNA assay using the virus‐containing dsDNA targets. Using the FQ assay, LbCas12a exhibited different cleavage efficiencies after crRNA recognition of its specific virus/viroid target (Figure S1). Among them, the crRNA of ACLSV showed the fastest dsDNA cleavage rate, but all reactions reached maximum fluorescence within 30 min.

**Figure 4:**
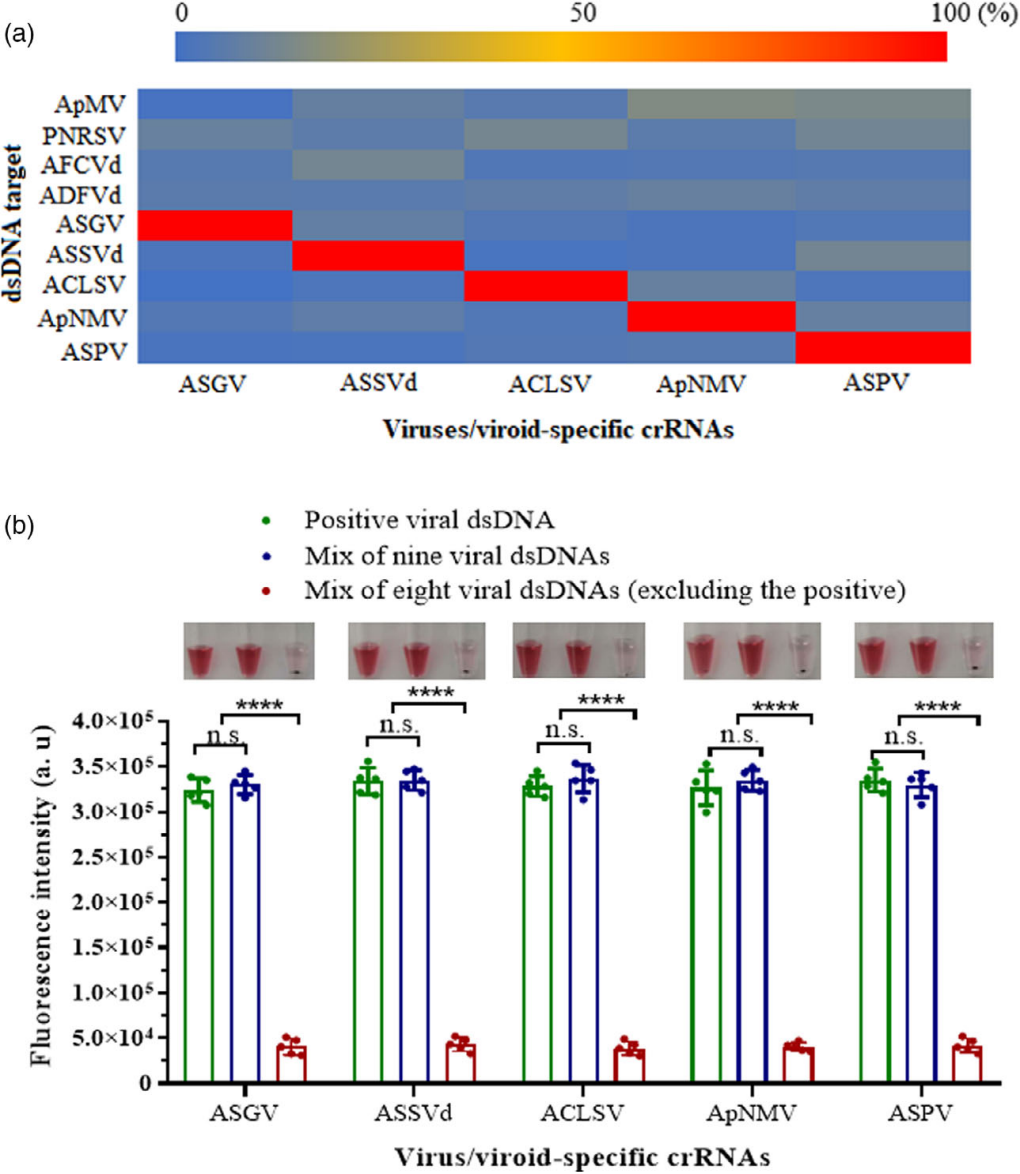
Evaluation of specificity for each virus/viroid‐targeting crRNA. (a) Heatmap showing the normalized mean fluorescence values after 30 min of LbCas12a reactions containing specific crRNAs (along the bottom) and dsDNA targets for the viruses/viroid ASPV, ASGV, ACLSV, ApNMV or ASSVd or non‐target dsDNAs for the viruses AFCVd, ADFVd, ApMV or PNRSV (left axis). (b) Virus/viroid‐specific fluorescence signals of the LbCas12a reactions at the 30‐min timepoint using different mixed targets. The photographs above present the colorimetric responses of the LbCas12a detection system containing the target viral dsDNA samples, nine viral dsDNAs, and all but the crRNA‐specific viral target after incubation with the DNA1/2‐AuNPs, with one tube from each set of five replicates shown. Background was not subtracted from the signal to show clear comparison between positive and negative outcomes. Data points represent replicates from five independent experiments, and the error bars indicate the mean ± SD. a. u., arbitrary unit. Asterisks indicate a significant difference with two‐tailed Student’s *t*‐test (****P < 0.0001). n.s., not statistically significant. [Colour figure can be viewed at wileyonlinelibrary.com]

The crRNAs showed good specificity for their respective targets, and there was no cross‐reactivity among the five targets (Figure 4a). Similarly, no significant fluorescence signals were detected from non‐target virus/viroid species (AFCVd, ADFVd, ApMV or PNRSV), which are found to co‐infect apple at low frequencies in China.

Samples that contained the positive target activated significant fluorescence signals and, once mixed with the DNA1/2‐AuNPs, maintained a stable red (Figure 4b). There was no significant fluorescence detected in reactions lacking the corresponding virus/viroid dsDNA, and after the addition of the DNA1/2‐AuNPs, the mixture lost its colour.

To more accurately evaluate the detection specificity, total cDNA from six leaf samples, RT‐PCR‐verified to be co‐infected with at least two viruses, were used as targets in assays as above (Figure S2A). One uninfected sample (sample 6 in Figure S2A; negative control) was included. The positive samples created higher fluorescence signals (without background subtraction) than the negative control after incubation for 1 h (Figure S2B). However, the amplitude of this signal was lower than the development reactions containing the positive virus dsDNA, even when up to 200 ng of cDNA was added to the reaction. In uninfected sample 6, only low background signals were generated. However, these levels of linker‐ssDNA degradation were insufficient to produce stable red colour when the reaction was mixed with the DNA1/2‐AuNPs (Figure S2B, upper pictures). Since viral loads have been documented at low copy numbers in apple (Ioanna *et al*., 2017), we speculated that the natural virus concentrations in a plant would not be enough to trigger sufficient nonspecific ssDNA cleavage by LbCas12a to result in a visible positive result. Accordingly, a pre‐amplification technique was tested within the system to increase the sensitivity of our diagnostic platform.

### Sensitivity assay for RT‐RPA/LbCas12a detection

The minimum detection limit of the LbCas12a/crRNA assays was initially determined using 10‐fold sequential dilutions of virus‐containing dsDNA. Obvious fluorescence signals, distinguishable from the background noise of negative controls, were observed when the concentration of the dsDNA target was 1 nm (ASSVd) or 0.1 nm (for the other four viruses), which translates to a limit of detection of around 2.5 × 10^8^–10^9^ viral copies per reaction (Figure S3). The apparent variation in the linker‐ssDNA cleavage efficiencies indicates that the crRNA targeting sequence plays an important role in determining the LbCas12a activity, thereby impacting the detection sensitivity.

When serially diluted *in vitro* RNA transcripts were used as the target, we incorporated an RT‐RPA pre‐amplification step into the diagnosis. RT‐RPA can convert viral RNA into dsDNA to further amplify viral targets. Evaluation of the specificity of the RT‐RPA primers for each virus/viroid showed that all pairs were specific only to their corresponding targets (Figure S4).

**Figure 5:**
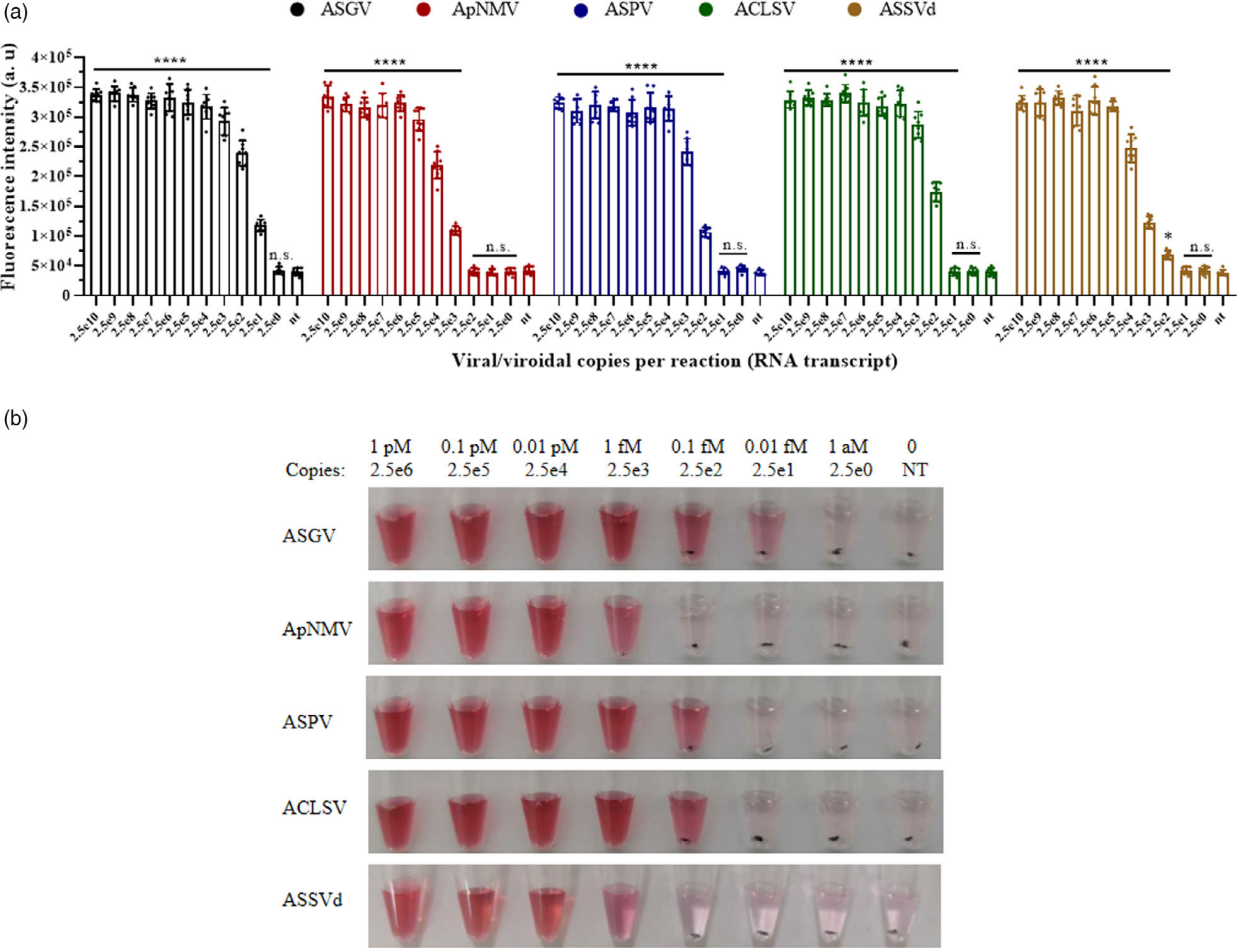
The sensitivity of the RT‐RPA/LbCas12a assay using fluorescence detection (a) and visual detection (b) for apple viruses/viroid. The *in vitro* RNA transcripts of each virus/viroid were amplified by RT‐RPA before being added to the LbCas12a/crRNA reaction. After 30 min of cleavage, the fluorescent signals (a) were detected in the virus/viroid‐specific LbCas12a/crRNA reactions. Fluorescence background was not subtracted from the signal to clearly compare the positive and negative outcomes. Data points represent replicates from eight independent experiments, and the error bars indicate the mean ± SD. a. u., arbitrary unit. The asterisk indicates a significant difference with two‐tailed Student’s *t*‐test compare to the control of no targets (*P < 0.05, ****P < 0.0001). n.s., not statistically significant. NT indicates no template control. (b) For the visual assay with DNA1/2‐AuNPs, one tube from each set of three replicates is shown. [Colour figure can be viewed at wileyonlinelibrary.com]

After a 20‐min reaction, RT‐RPA products were applied to the LbCas12a/crRNA fluorescence detection assay. Extremely high fluorescence (*P* < 0.0001) was generated when the viral/viroid copies per reaction were as low as 25 for ASGV, 250 for ASPV and ACLSV, and 2500 for ApNMV and ASSVd (Figure 5a). This translates to a detection limit of RNA transcripts around 0.01–1 fM. The variations in the LODs are probably due to RT‐RPA efficiencies and LbCas12a activity triggered by diverse targets. These LbCas12a test reactions were mixed with the DNA1/2‐AuNPs, and stable red solutions were observed with comparable sensitivity as the fluorescence assay (Figure 5b). From these assays, we concluded that the LOD of the integrated RT‐RPA/Cas12a/AuNP assay for an RNA virus/viroid could be as low as 25 viral copies per reaction when using optimized RPA primers or target sequences.

### Development of multiplex RT‐RPA reactions coupled with parallel LbCas12a/crRNA cleavage reactions and AuNP visual detection

Successful operation of the single‐plex RT‐RPA combined with LbCas12a/AuNP visual detection encouraged us to try multiplex RT‐RPA by combining five sets of virus/viroid‐specific primers in a single tube. First, various primer concentrations were optimized for the multiplex experiments. All of the targets were pre‐amplified with multiplex RT‐RPA from serial dilutions of mixed RNA transcripts containing each virus/viroid. The RT‐RPA products were then added to individual tubes for each LbCas12a/crRNA reaction (LbCas12a coupled with virus/viroid‐specific crRNA) and fluorescence detection (Figure S5A‐C). Using the appropriate primer concentration combinations, we found that the sensitivity was reduced when target concentrations were at or lower than 2.5 × 10^2^ viral copies per reaction for ASPV and ASGV and than 2.5 × 10^3^ for the others (Figure S5C). A further optimization for the primer concentrations or other characteristics might improve the amplification efficiency of the multiplex RT‐RPA and thus enhance analytical sensitivity.

**Figure 6:**
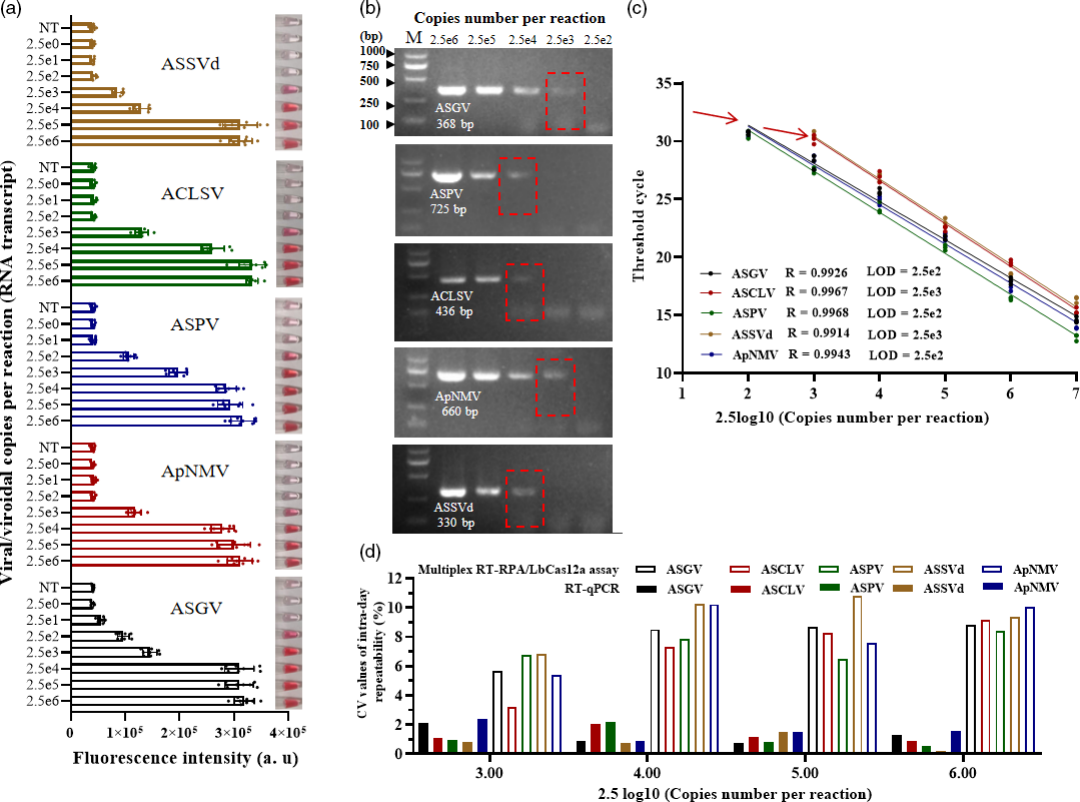
Sensitivity and repeatability comparison of three methods for detecting four viruses and one viroid using 10‐fold serial dilutions of RNA transcripts. (a) Sensitivity of the multiplex RT‐RPA/LbCas12a/AuNP assays. Fluorsecence detection and one tube from each set of three replicates is shown. (b) Sensitivity of conventional RT‐PCR, amplified with specific primers. Viral/viroid copy numbers are indicated above the lanes. M, DL2000 DNA ladder. (c) Standard curve for one‐step TaqMan RT‐qPCR amplification of each RNA transcript. The obtained threshold cycle for the dilutions was used for a linear regression with the viral/viroid copy numbers. The red arrows indicate the minimum number of RNA transcripts that can be detected by each standard curve. The sensitivity was determined to be 2.5 × 10^2^ viral copies per reaction for ASPV and ASGV and of 2.5 × 10^3^ for ApNMV, ACLSV and ASSVd. (d) Intra‐day repeatability comparison of RT‐qPCR (empty bars) and multiplex RT‐RPA/LbCas12a fluorescence assays (solid bars) using coefficient of variation (CV). [Colour figure can be viewed at wileyonlinelibrary.com]

For comparison, serial dilutions of *in vitro* RNA transcripts for each virus/viroid were prepared and subjected to one‐step RT‐PCR and TaqMan RT‐qPCR assays. The multiplex RT‐RPA/LbCas12a/AuNP assay for simultaneous detection of five virus/viroid targets was 10‐100 times more sensitive than RT‐PCR detection (Figure 6a‐b; Table S1). Sensitivity of this method was the same as that of the TaqMan RT‐qPCR assay for the detection of ASGV, ASPV, ACLSV and ASSVd, but was slightly less for the detection of ApNMV (Figure 6c). The single‐plex RT‐RPA/LbCas12a/ AuNP assay showed higher sensitivity when detecting ASGV and ACLSV compared with RT‐qPCR results (Table S1; Figure 5b). For each method, intra‐day repeatability and inter‐day reproducibility, which were expressed as the coefficient of variation (CV), were also evaluated at four concentration levels (2.5 × 10^3^–10^6^ viral copies per reaction) over six days by performing assays eight times each day. The CV values for multiplex RT‐RPA/LbCas12a fluorescence assays ranged from 3.2% to 10.7% for intra‐day repeatability (Figure 6d) and 6.04 to 11.97% for inter‐day reproducibility (Figure S6). These CV values were relatively higher than those of TaqMan RT‐qPCR, but still indicated a repeatability similar to that reported for SHERLOCK (Gootenberg *et al*., 2017). Although our detecting platform exhibited comparable sensitivities and slightly higher variation than TaqMan RT‐qPCR, the protocol required to conduct a multiplex RT‐RPA/LbCas12a assay is significantly simpler, less expensive and faster (1h) compared to those required to perform RT‐qPCR (2 h).

**Figure 7:**
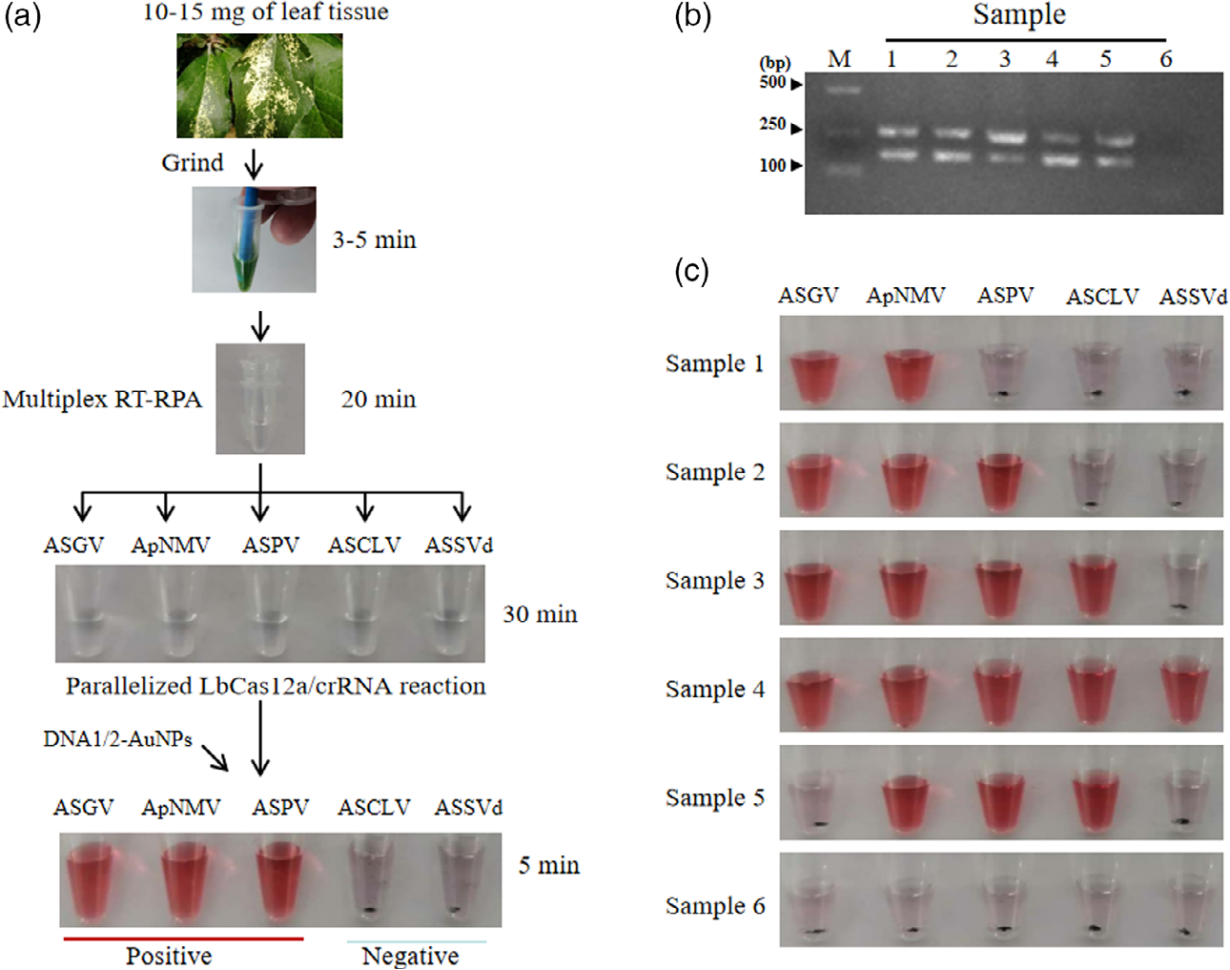
Parallel detection of four RNA viruses and one viroid using multiplex RT‐RPA/LbCas12a/AuNP visual assay. (a) Schematic representation of detection process. RT‐RPA with five sets of primers is carried out in one tube. (b) Image of agarose gel following electrophoresis of multiplex RT‐RPA products using the total RNA extracted from six apple leaves. (c) Parallel visual detection of five viral targets using LbCas12a/AuNP assays on the RT‐RPA products from (b). One tube from each set of three replicates is shown. [Colour figure can be viewed at wileyonlinelibrary.com]

In addition to the single‐tube multiplex RT‐RPA protocol, we established a simplified RNA isolation method for in‐field preparation of samples using a hand‐held tissue homogenizer. Moreover, the incubations for the multiplex RT‐RPA and the LbCas12a/crRNA reaction were carried out at body temperature (36–37 °C), be being held in the hands. The entire protocol, from harvesting the sample, requires no more than an hour to perform (Figure 7a).

This protocol accomplishes fast preparation and detection in the field. The extraction of viral RNA is efficient. Subjecting the crude leaf extracts of virus‐infected samples to amplification using the multiplexed RT‐RPA assays produced only two, clearly distinguishable, bands following gel electrophoresis (Figure 7b). These bands likely contain two sets of similarly sized amplification products (165, 157 bp and 255, 275 bp). However, specific positive results for each virus/viroid were detected and differentiated from the amplification products by subsequent LbCas12a/AuNP visual assays, indicating that all targets were successfully amplified by multiplex RT‐RPA (Figure 7c). When 10‐fold serial dilutions of the crude extracts were used in the assay, we detected ASGV and ASPV up through a 10^−2^ dilution and the others up through a 10^−1^ dilution (Figure S7). These promising results indicate that our approach might also run in more complex crude samples.

In a final validation, we collected a total of 52 samples from commercial orchards located in different parts of Henan Province, China, to determine the efficacy of the new method using only microfuge tubes, a hand‐held tissue homogenizer and a micropipette (Finnpipette, Fisher Scientific). All commercial samples were virus positive, at the following rates: 67.3% positive for ASGV, 63.5% for ACLSV, 57.7% for ASPV, 78.8% for ApNMV and 32.7% for ASSVd (Figure S8; Table S2). Infections of at least three viruses were common. The most frequent virus combinations were ASGV + ASPV + ApNMV, with an incidence of 17.31%, followed by ASGV + ACLSV+ApNMV (13.46%) and ASGV + ACLSV + ApNMV + ASSVd (11.54%) (Table S3). To confirm the results using the newly developed assay, RT‐PCR was performed after RNA was extracted in a traditional way from those samples. The results were consistent with the RT‐PRA/Cas12a/AuNP analysis, indicating an excellent diagnostic agreement between the two methods (Table S2).

## Discussion

A simple‐to‐use diagnostic test that rapidly and reliably detects virus infection of saplings or fruit trees in the field could facilitate early treatment, inhibiting the spread of viral diseases. However, current RNA virus diagnostics for fruit trees, such as RT‐PCR (Ji *et al*., 2013) or next generation sequencing (Jones *et al*., 2017), can only be performed in laboratory settings, which causes a significant delay between sample collection and test results during quarantine. In the present study, we described a CRISPR/LbCas12a‐based method, comprising, firstly, a multiplex RT‐RPA reaction, in which four RNA viruses and one viroid can be rapidly amplified concurrently in one tube using only crude leaf extracts, and, secondly, highly specific LbCas12a/AuNP assays with rapid visual readout. This method can be performed at body temperature without the need for a laboratory environment, and the entire process from sample preparation to result takes only an hour. These factors make it amenable to field diagnostics, thus eliminating the need to transport samples and to wait for analysis. This robust and field‐based diagnostic method can be potentially used to assist in the production and certification of virus‐free saplings and to move plants through quarantines.

RT‐RPA is more robust than RT‐PCR when naturally occurring inhibitors are present within the sample, and thus can accurately detect RNA virus from crude plant extracts without more complex nucleic acid extraction protocols. However, it is still possible to encounter nonspecific amplification (Chaijarasphong *et al*., 2019; Mayboroda *et al*., 2018). Furthermore, amplification products of similar size from multiplexed targets are difficult to separate by electrophoresis in agarose gels. This drawback may be overcome by real‐time monitoring with exo probes (Ahmed *et al*., 2020), or by visualization with lateral flow dipstick and nfo probes (Rames and Macdonald, 2019), but the need for probes restricts this method due to the complexity of probe design (TwistDx, 2018). Our method involves a second step of specific LbCas12a/crRNA recognition of amplicons that contain the target sequence. This step excludes spurious amplicons, allowing the overall method to benefit from the high tolerance to inhibitors for which RPA is known.

Two other recently characterized CRISPR nucleases, Cas13 (Gootenberg *et al*., 2017) and Cas14 (Harrington *et al*., 2018), have been developed for nucleic acid detection. The RNase activity of Cas13a/crRNA can be triggered by ssRNA targets and can cleave nearby non‐target RNA reporters. Cas13a/crRNA has been developed into a platform for the detection of a variety of RNA viruses, such as Zika virus (Gootenberg *et al*., 2017), dengue virus (Myhrvold *et al*., 2018) and avian influenza A (H7N9) virus (Liu *et al*., 2019). However, Cas13a‐based detection still relies on both the pre‐amplification of viral RNA by RT‐RPA and an additional step of in vitro transcription that converts dsDNA amplicons into RNA targets. Additionally, the RNA reporters are susceptible to degradation by ubiquitous RNases, which can cause false negatives in field testing (Sashital, 2018). In comparison, Cas12a/crRNA can directly detect dsDNA amplicons, and both the DNA reporter described in the DETECTR (Chen *et al*., 2018) and HOLMES (Li *et al*., 2018b) systems and the linker‐ssDNA used in our method are more stable. Although most groups use the Cas12a system to detect dsDNA targets (Chaijarasphong *et al*., 2019; Li *et al*., 2018a; Li et al., 2019; Wang *et al*., 2019), RNA targets can also be detected if RNA is reverse‐transcribed into complementary DNA (cDNA) following the RT‐RPA or RT‐LAMP reaction (Broughton *et al*., 2020). Cas14 has a functional similarity to Cas12a, but Cas14‐based DNA tests require extra steps to generate the targeting ssDNA substrate from dsDNA amplicons, which is also relatively complicated (Harrington *et al*., 2018). Since these three systems have similar sensitivities and specificities, we believe that Cas12a is a more effective tool for detecting both RNA and DNA targets.

Multiplex detection of RNA viruses in a single reaction makes it possible to gain more information from samples suspected to be infected with viruses and to reduce time and costs. So far, most CRISPR‐based assay platforms are limited to detecting only one target sequence (Chaijarasphong *et al*., 2019; Li et al., 2019; Wang *et al*., 2019; Zhang *et al*., 2020a). The SHERLOCK version 2 platform could simultaneously detect four targets via four‐channel single‐reaction multiplexing with orthogonal CRISPR enzymes (Gootenberg *et al*., 2018). However, this requires four fluorescent reporters and relies on fluorescence detection equipment for the readout, which is expensive and limits its application in the field.

In this study, we proposed a scheme involving pre‐amplification of virus sequences with multiplex RT‐RPA, followed by concurrent detection of RNA viruses/viroid using separate LbCas12a/AuNP visual assays in parallel. Although this method is not truly a one‐tube multiplex detection and requires additional steps of transferring amplification products to the different tubes for the virus/viroid‐specific Cas12a/crRNA step, it can achieve simultaneous detection of five targets without the need for complex equipment or precise temperatures. In addition, we used gold nanoparticles for the visual readout, allowing direct observation of the result in the tube. This method is more cost‐effective than other visual strategies, such as lateral flow strips (Zhang *et al*., 2020b) or fluorescent signals excited under blue light (Wang *et al*., 2019), because it does not require expensive FQ‐labelled probes or the commercial lateral flow strips. Furthermore, NaCl solution was introduced to accelerate the irreversible aggregation and colour transition of DNA1/2‐AuNP, which is more easily implemented in the field. In the future, it might also be possible to develop multiplexed visual readouts for detecting different targets in one tube.

In conclusion, we have developed a potentially field‐deployable method for simultaneous detection of four RNA viruses and one viroid in apple, based on the integrated RT‐RPA/Cas12a/AuNPs protocol. With minimal sample preparation requirements, we could detect each RNA virus/viroid directly from the crude leaf extracts with high sensitivity and specificity, not requiring technical expertise and specialized equipment. As the method is highly simplified, it provides a promising alternative to currently used laboratory‐based diagnostic techniques. Overall, this CRISPR‐Cas12a‐based method has the potential to be used in certification facilities to assist in the rapid confirmation of virus‐infected apple plants or screening for virus‐free saplings.

## Experimental methods

### Sample collection, RNA isolation and virus detection by RT‐PCR

Apple leaves were collected from commercial orchards in the major apple‐growing regions of Luoyang and LingBao city in Henan Province, China, over the summer of 2019. The selected orchards were more than ten years old. Both symptomatic and asymptomatic leaves were collected randomly from different cultivars. Total RNA was extracted from 0.5 g of each sample with TRIzol reagent (Tiangen, Beijing, China), followed by first‐strand cDNA synthesis using the PrimeScript RT Reagent Kit with gDNA Eraser (Takara, Dalian, China). Conventional RT‐PCR with virus/viroid‐specific primers (Table S4) was performed to define the disease state of each sample. RT‐PCR was conducted using RNA from virus‐free apple plantlets, which were a gift from Prof. Yanxiu Wang, Gansu Agricultural University, as negative controls for ASPV, ASGV, ACLSV, ASSVd and ApNMV.

### RT‐RPA primer and crRNA design

Two complementary oligonucleotides, NTS‐40 and TS‐40 (Table S5), were annealed to form a 40‐nt target dsDNA (target‐40). Paired oligonucleotides (1 µm) were annealed in 1 × annealing buffer (Solarbio, Beijing, China) in a 20‐µL reaction and then subjected to an initial denaturation at 95 °C for 2 min and then a cool down by 1 °C per min to 20 °C using a thermocycler.

For each virus/viroid, RT‐RPA primers were designed from the conserved region following multiple sequence alignment of the available virus sequences in the GenBank database, using the criteria suggested in the TwistAmp reaction kit manual. A BLAST search, against all the genomic sequences deposited in the NCBI database, was carried out to check the specificity of each RT‐RPA primer pair.

We designed crRNAs to recognize a site specific to each virus/viroid and to have homology to the region within an amplicon between the RT‐RPA primer pairs (Figures [Link], [Link], [Link], [Link], [Link]). The five crRNAs recognize a 20‐bp target sequence adjacent to a TTTN or NAAA site and were designed from the highly conserved region of each virus/viroid as found in the NCBI. The chosen crRNA sequences were then checked against the available sequences in the NCBI GenBank database using the BLAST program to ensure that there was no highly homologous sequence elsewhere.

### Generation of RNA standards

A genomic fragment harbouring the target sequences of the RT‐RPA primers and crRNA was PCR‐amplified from the total cDNA of virus‐infected leaves with the RT‐RPA primers, sub‐cloned into the pGEM‐T vector (Promega), and verified by sequencing. The pUC/M13 universal primers (Promega) were used to amplify the positive inserts. These second PCR products were then diluted to 100 ng/µL and were employed as the dsDNA targets for each viral sequence for preliminary experiments. RNA targets of each virus/viroid were generated from the purified virus‐containing dsDNA, using the HiScribe T7 Quick High Yield RNA Synthesis kit (New England Biolabs). The *in vitro*‐transcribed RNA was quantified, serially diluted to concentrations ranging from 10 nm to 10^−9^ nm and converted to copy number.

The RNA transcripts used for the RT‐PCR and RT‐qPCR assay were also synthesized *in vitro* as above, with the individual primer sets listed in Table S4 and Table S6.

### crRNA synthesis

DNA templates encoding crRNAs were prepared through annealing of the synthesized oligonucleotides with T7‐crRNA‐F (Table S7), as previously described (Li *et al*., 2017). The crRNAs were transcribed using a TranscriptAid T7 High Yield Transcription Kit, according to the manufacturer’s protocol (Thermo Fisher Scientific). After purification with the RNA Clean & Concentrator^™^‐5 (Zymo Research), crRNAs were quantified with a NanoDrop 2000 (Thermo Fisher Scientific), diluted to 10 µm and stored at −80 ºC until further use. The target efficiencies of each crRNA were evaluated by Cpf1‐CRISPR‐DT online software (http://bioinfolab.miamioh.edu/CRISPR‐DT/interface/Cpf1_main.php).

### Preparation of DNA functionalized AuNPs

Two DNA functionalized AuNPs were prepared using an mPEG‐SH/Tween‐20‐assisted method, as reported (Li *et al*., 2014). Briefly, 1 mL of 13‐nm AuNP solution (2.5 nm) was first mixed with 10 µL of 1% Tween‐20 and 50 µL of 4 µm mPEG‐SH (MW ~ 5 kDa). Then, 10 µL of 100 µm thiolated poly(A)‐tagged DNA1 (GTGTTGCTTGTAGTATTTTTAAAAAAAAAA‐SH) or DNA2 (GTGTTTAGGATTTGCTTTTTAAAAAAAAAA‐SH) was added to the solution, along with 4 m NaCl to a final concentration of 800 mm. After 1 h at room temperature, the free DNA was removed by centrifugation for 10 min at 12 800**g**. The pellet containing the DNA‐nanoparticle was washed three times with 1 mL PBS buffer (pH 7.4). The two DNA‐AuNPs (DNA1 and DNA2) were separately dissolved into 0.5 mL PBS buffer containing 0.01% Tween‐20 and stored at 4 °C.

### Optimization of the experimental condition for Cas12a/crRNA cleavage assay and AuNP‐based visual detection

The dose of linker‐ssDNA was optimized for the AuNP‐based visual detection. The assay was performed with 1 nm DNA1‐AuNP, 1 nm DNA2‐AuNP and varied concentrations of linker‐ssDNA (TCCTAAACACCACAACGAAC) in a 50‐μL reaction. To accelerate the irreversible aggregation of the AuNPs with the linker‐ssDNA, 5 µL of saturated NaCl solution was added to the reaction. After incubation at room temperature for 1 min and 5 min, the absorbance of the mixture was scanned from 450 nm to 700 nm using a spectrophotometer (Mapada V‐1800, Shanghai, China). The final concentration of linker‐ssDNA was set to 50 nm for future assays.

Effects of buffer type, the concentration of Cas12a‐crRNA complexes and incubation time were investigated in the Cas12a/crRNA cleavage assay. The Cas12a nuclease of *Lachnospiraceae bacterium* ND2006 (LbCas12a) was purchased from New England Biolabs Ltd. (Whitby, ON, Canada). LbCas12a‐crRNA complexes were pre‐assembled by incubating 400 nm LbCas12a and 500 nm crRNA at 37 °C for 5 min. The assays were performed with 10 nm target‐40, 100 nm fluorophore quencher (FQ)‐labelled linker‐ssDNA (HEX‐TCCTAAACACCACAACGAAC‐BHQ1), 10 U of RNase inhibitor, different buffers [NEBuffer 2.1, NEBuffer 3, NEBuffer 3.1, and 1 × binding buffer (Chen *et al*., 2018)], and varying concentrations of LbCas12a‐crRNA complexes in a 25‐µL volume. Reactions were carried out at 37 °C for 30 min. Fluorescence was excited at 535 nm and detected at 556 nm using a Bio‐Tek FLx800 microplate fluorescence reader. The background‐corrected fluorescence values were measured by subtracting background fluorescence obtained from the negative control without targets. The resulting solution was mixed with an equal volume of a premixed DNA1/2‐AuNP solution (2 nm for each), followed by the colorimetric detection performed as above. The experiment was repeated five times.

### Evaluation of specificity for virus/viroid‐targeting crRNAs

Complete nucleotide sequences of ASGV, ASPV, ACLSV, ASSVd and ApNMV were obtained by long‐distance PCR from virus/viroid‐infected apple leaves as described previously (Dhir et al., 2014; Puchta *et al*., 1990; Xing *et al*., 2018). To demonstrate the assay specificity, the related non‐target viroids and viruses, namely *Apple fruit crinkle viroid* (AFCVd, accession no. AB429195), *Apple dimple fruit viroid* (ADFVd, accession no. KC237730), RNAs 1 to 3 genome sequences of *Apple mosaic virus* (ApMV, accession nos. NC003464, NC003465 and NC003480) and *Prunus necrotic ring spot virus* (PNRSV, accession nos. NC004362, NC004363 and NC004364), were synthesized by GENEWIZ (Suzhou, China) and tested as follows.

Under the optimum conditions, preliminary specificity of the virus/viroid‐targeting crRNAs was screened by a LbCas12a‐based fluorescence assay with different dsDNA targets: (i) 10 nm of the virus/viroid‐containing dsDNA; (ii) a mixed sample containing 10 nm of dsDNA targets for each virus/viroid; (iii) a mixed sample containing 10 nm of dsDNA targets for each virus/viroid excluding the one corresponding to the crRNA being tested; and (iv) total cDNA from several RT‐PCR‐verified virus‐infected apple leaves. Total cDNA from virus‐free plantlets was included in each assay as a negative control and was also added into samples (i)‐(iii) to mimic the sample complexity. Unless otherwise described, the integrated LbCas12a/crRNA fluorescence and visualization detection procedures were as follows: the optimized assays were performed with 50 nm LbCas12a, 62.5 nm crRNA, 100 nm of (FQ)‐labelled linker‐ssDNA, 2.5 μL of nucleic acid target and add NEBuffer 2.1 up to 25 μL. Reactions were carried out at 37 °C for 30 min, and the fluorescence values were detected as above. After fluorescence detection, 25 μL of the DNA1/2‐AuNP solution and 5 µL of a saturated NaCl solution were added to each tube for colorimetric detection as above.

### Sensitivity of the assay

The dsDNA targets of each virus/viroid (10 nm) were serially diluted 10‐fold (1.0 to 1.0 × 10^−10^) and subjected to LbCas12a/crRNA fluorescence and visualization as described above to determine the limit of detection (LOD).

To test the sensitivity of single‐plex or multiplex RT‐RPA/LbCas12a/crRNA assay, the RNA transcripts of each virus/viroid (10 nm) were also serially diluted 10‐fold (1.0 to 1.0 × 10^‐10^) with DEPC‐treated water. Each dilution was amplified prior to the LbCas12a‐based fluorescence assay by RT‐RPA (see below). After incubation at 37 °C for 20 min, 2.5 µL of product was then used for Cas12a/crRNA detection as described above. The RT‐RPA products were also purified and analysed by gel electrophoresis. To determine the LOD, we used Student’s *t*‐test to compare each result with the fluorescence of the no‐target control.

### Isothermal amplification by RPA

RT‐RPA was performed with TwistAmp Basic (TwistDx, United Kingdom) and HiScript III Reverse Transcriptase (Vazyme, Nanjing, China), which has an optimum temperature range of 37–42 °C and is compatible with RPA reagents, to convert the viral RNA into cDNA (Lillis *et al*., 2016). All single‐plex and multiplex assays were performed in duplicate using the primers listed in Table S6.

For single‐plex RT‐RPA assays, reactions were performed in a 50‐μL volume with some modifications to the manufacturer’s protocol. Briefly, the enzyme pellets of the kit were first hydrated with 2.5 µL of each primer (10 µm; Table S8), 2.5 µL HiScript III Reverse Transcriptase (Vazyme, Nanjing, China; 200 U/µL), 2.5 µL RNase inhibitor (Tiangen, Beijing, China; 40 U/µL), 29.5 µL rehydration buffer, 3 µL RNase‐free water and 5 µL RNA (total RNA of leaves, varying concentrations of RNA transcripts or crude leaf extracts). The last 2.5 μL of 280 mm magnesium acetate was added into the tube lid and then spun down to initiate the reaction. After incubation at 37 °C for 20 min, 2 µL of product was used for either Cas12a fluorescence or AuNP visual detection with individual virus/viroid‐specific crRNA, unless otherwise described.

For multiplex RT‐RPA assays, the optimized concentrations of both forward and reverse primers for each virus/viroid were adjusted to the following: 100 nm for ASGV and ASPV; 150 nm for ACLSV; and 200 nm for ApNMV and ASSVd, such that the total oligonucleotide concentration in one reaction was 1500 nm. Moreover, 0.8 m betaine (Sigma Aldrich, UK) was added to the reaction mixture to improve the sensitivity and specificity (Luo *et al*., 2019).

### One‐step RT‐PCR and TaqMan RT‐qPCR assay

The newly developed multiplex RT‐RPA/LbCas12a/AuNP visual assay was compared with two other established methods, one‐step RT‐PCR and TaqMan RT‐qPCR. The transcribed RNA standards were 10‐fold serially diluted by DEPC‐treated water and used to evaluate the sensitivity. One‐step RT‐PCR was first performed using a PrimeScript™ One‐Step RT‐PCR Kit (Takara, China) with virus/viroid‐specific primers (Table S4), according to the manufacturer’s protocol. PCR products were analysed by gel electrophoresis to determine the LOD. Next, TaqMan‐based RT‐qPCR assays were carried out using a HiScript II One‐Step qRT‐PCR Probe Kit (Vazyme, China) with the TaqMan‐MGB probe and primer set listed in Table S6. Assays were performed using a LightCycler480 (Roche). The RT‐qPCR profile consisted of cDNA synthesis (15 min at 50 °C), an initial denaturation step (30 s at 95 °C) and 45 amplification cycles (10 s at 95 °C, 30 s at 60 °C). Fluorescence information was collected during the 60 °C annealing step per cycle. Each dilution was tested in three replicates to identify an endpoint beyond which test positivity was unattainable. Sensitivity and linearity of the RT‐qPCR assays were estimated by constructing standard curves.

### Direct virus detection from crude extracts

For crude leaf extraction, a modified alkaline polyethylene glycol (PEG) extraction method was used as previously described (Silva *et al*., 2018). Briefly, 15–20 mg of leaf tissue was homogenized by a hand‐held tissue homogenizer in a 1.5‐mL tube with 300 μL extraction buffer (6% PEG 200 and 20 mm NaOH). After incubation at room temperature for 3–5 min, the crude extract was directly subjected to RT‐RPA without purification, followed by LbCas12a/AuNP visual detection using the above procedure.

The crude extract above was considered the 1x dilution. Further 10‐fold serial dilutions, 10^1^, 10^2^, 10^3^, 10^4^ and 10^5^, were made and tested. Validation and comparative analysis were performed on 52 plant samples derived from commercial orchards located in different parts of Henan, China.

## Conflict of Interest

The authors declare that the research was conducted in the absence of any commercial or financial relationships that could be construed as a potential conflict of interest.

## Author contributions

JJ, KKK and JMH designed and performed experiments and co‐wrote the manuscript. JJ and KKK contributed equally to this work. JCF and XBZ conceived this project and supervised the experiments. SWS, THB, CHS and MMW analysed the data. ZLY, HTZ and RPZ contributed apple materials and reviewed the manuscript. All authors read and approved the final manuscript.

## Supporting information


**Figure S1** Time course of fluorescence detection of the LbCas12a/crRNA reactions with the corresponding targets.

**Figure S2** Identification of four apple viruses and one viroid in six samples by RT‐PCR (A) and LbCas12a‐mediated cleavage of a fluorescent quencher without nucleic acid amplification (B).

**Figure S3** Comparison of the sensitivity of direct LbCas12a fluorescence detection for four RNA viruses and one viroid without nucleic acid amplification.

**Figure S4** Specificity of RT‐RPA amplification of four RNA viruses and one viroid using total RNA isolated from several co‐infected samples.

**Figure S5** Fluorescence values detecting four RNA viruses and one viroid by multiplex RT‐RPA followed by LbCas12a/crRNA assay using five primer pairs at different concentrations and different target DNA concentrations.

**Figure S6** Inter‐day reproducibility comparison of RT‐qPCR and multiplex RT‐RPA/LbCas12a fluorescence assays using coefficient of variation.

**Figure S7** Detection of four RNA viruses and one viroid by multiplex RT‐RPA/LbCas12a/AuNP assays using serial dilutions of a crude extract.

**Figure S8** Detection of four RNA viruses and one viroid in 52 apple leaf samples using the RT‐PRA/LbCas12a/AuNP assay.

**Figure S9** Design of the specific crRNA for ASGV detection.

**Figure S10** Design of the specific crRNA for ApNMV detection.

**Figure S11** Design of the specific crRNA for ACLSV detection.

**Figure S12** Design of the specific crRNA for ASSVd detection.

**Figure S13** Design of the specific crRNA for ASPV detection.

**Table S1** Comparison of sensitivity among virus/virod detection assays with each of serially diluted RNA standards.
**Table S2** Comparison between RT‐PCR and RT‐RPA/LbCas12a/AuNP for the detection of four RNA viruses and one viroid in apple leaves collected from different apple varieties from commercial orchards in different regions within Henan Province.
**Table S3** Number of mixed virus/viroid‐infections in samples collected from commercial fields as detected by the multiplex RT‐RPA/LbCas12a/AuNP visual assay.
**Table S4** Specific primers for virus/viroid detection by RT‐PCR.
**Table S5** Targeted sequences for crRNAs and RPA primers
**Table S6** The primers and TaqMan‐MGB probes used in the one‐step RT‐qPCR assay.
**Table S7** Oligonucleotides used for preparing transcription templates of crRNAs.
**Table S8** RT‐RPA primers designed in this study.
