# Supplementary figures and images for: Field detection of multiple RNA viruses/viroids in apple using a CRISPR/Cas12a‐based visual assay
Source: Plant Biotechnol J. 2020 Sep 17;19(2):394–405. doi: 10.1111/pbi.13474 (PMC7868969; doi:10.1111/pbi.13474)

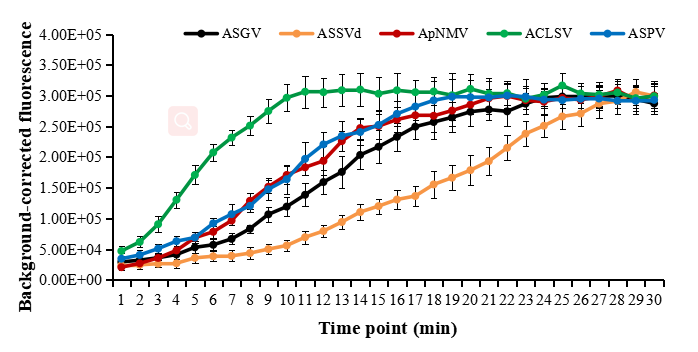

Supplement: Supplementary file 1 — Figure S1 Time course of fluorescence detection of the LbCas12a/crRNA reactions with the corresponding targets. [file PBI-19-394-s004.png]

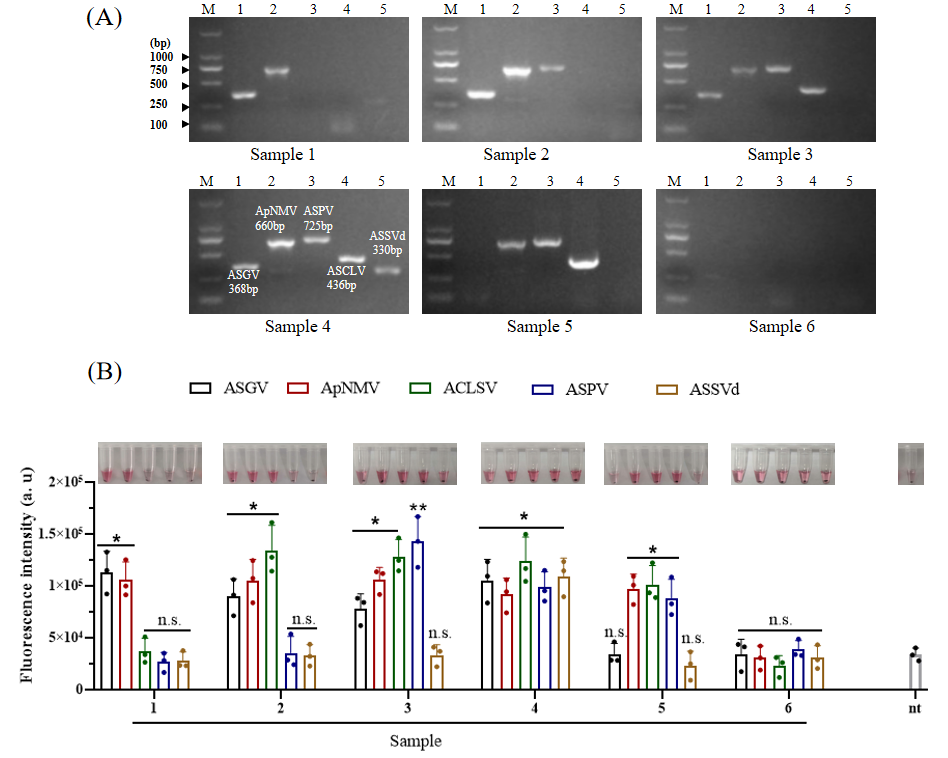

Supplement: Supplementary file 2 — Figure S2 Identification of four apple viruses and one viroid in six samples by RT‐PCR (A) and LbCas12a‐mediated cleavage of a fluorescent quencher without nucleic acid amplification (B). [file PBI-19-394-s002.png]

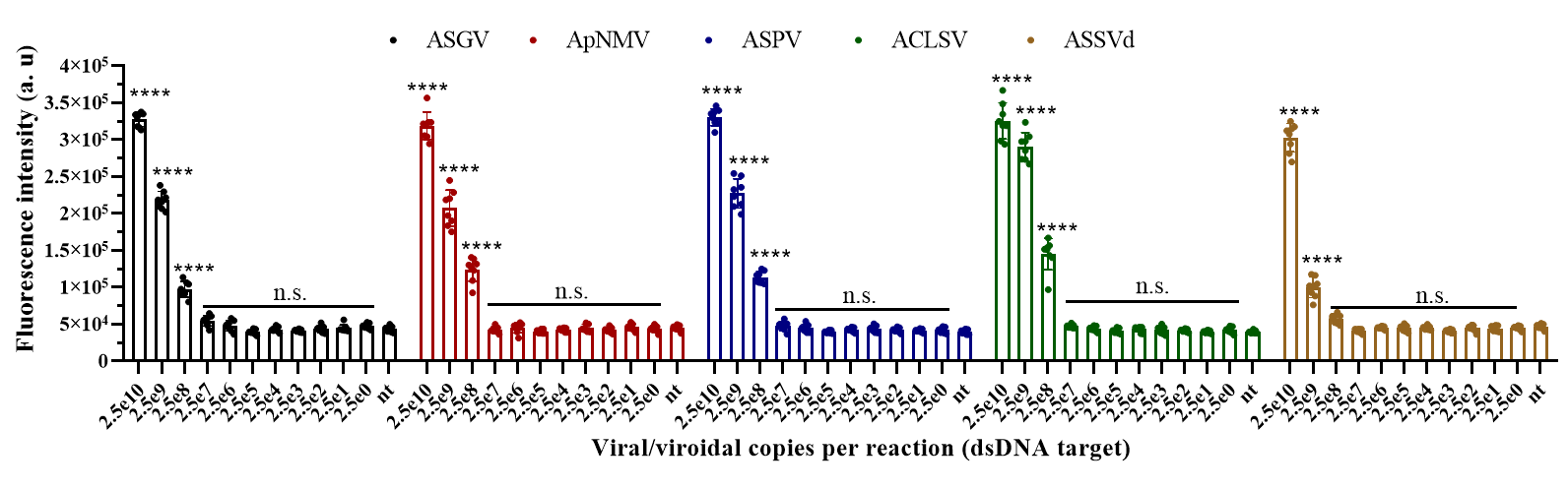

Supplement: Supplementary file 3 — Figure S3 Comparison of the sensitivity of direct LbCas12a fluorescence detection for four RNA viruses and one viroid without nucleic acid amplification. [file PBI-19-394-s007.png]

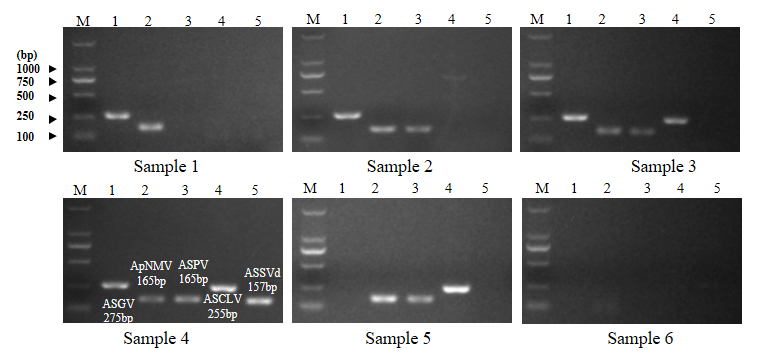

Supplement: Supplementary file 4 — Figure S4 Specificity of RT‐RPA amplification of four RNA viruses and one viroid using total RNA isolated from several co‐infected samples. [file PBI-19-394-s001.png]

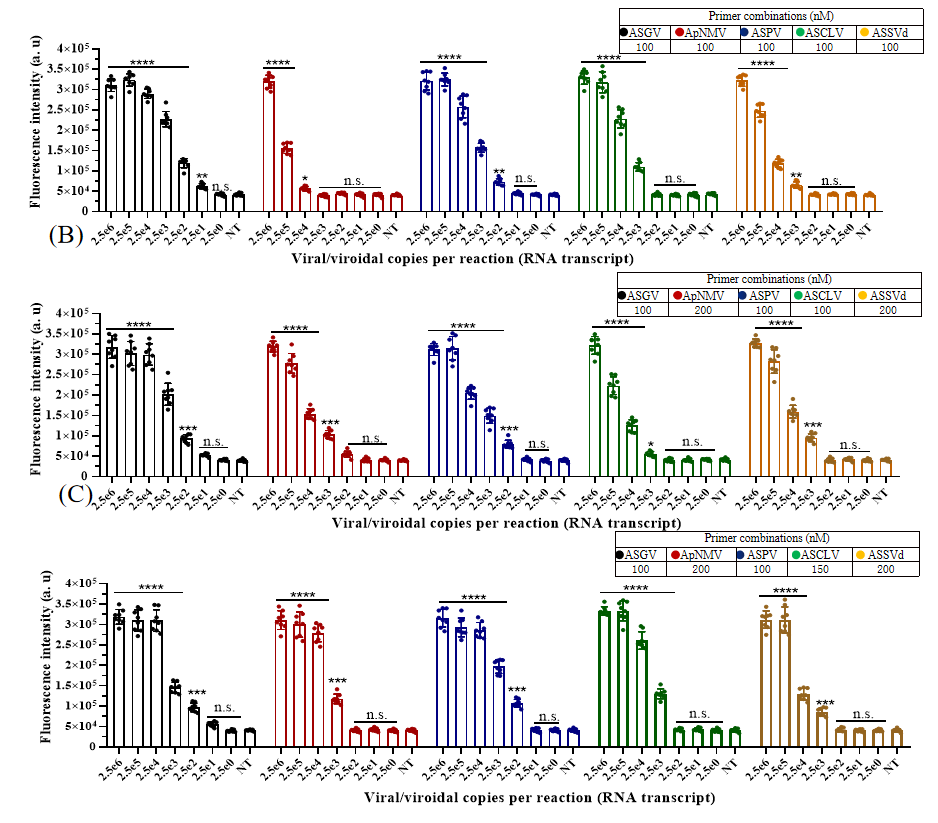

Supplement: Supplementary file 5 — Figure S5 Fluorescence values detecting four RNA viruses and one viroid by multiplex RT‐RPA followed by LbCas12a/crRNA assay using five primer pairs at different concentrations and different target DNA concentrations. [file PBI-19-394-s005.png]

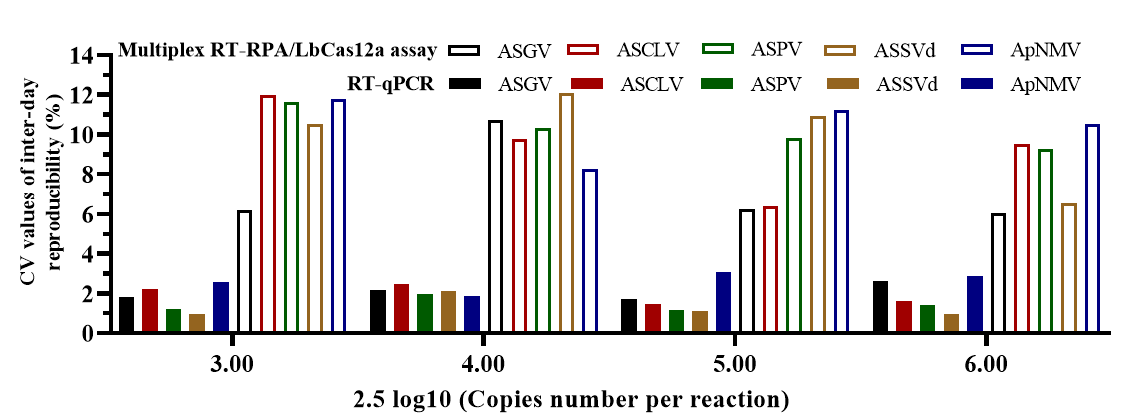

Supplement: Supplementary file 6 — Figure S6 Inter‐day reproducibility comparison of RT‐qPCR and multiplex RT‐RPA/LbCas12a fluorescence assays using coefficient of variation. [file PBI-19-394-s003.png]

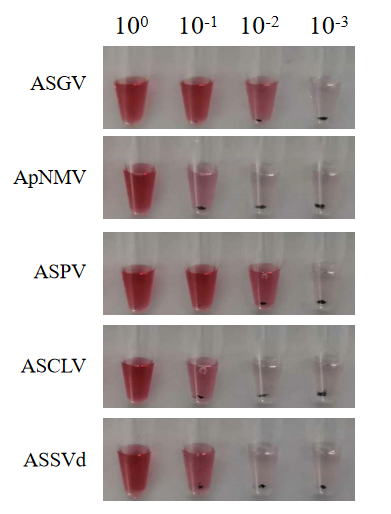

Supplement: Supplementary file 7 — Figure S7 Detection of four RNA viruses and one viroid by multiplex RT‐RPA/LbCas12a/AuNP assays using serial dilutions of a crude extract. [file PBI-19-394-s014.png]

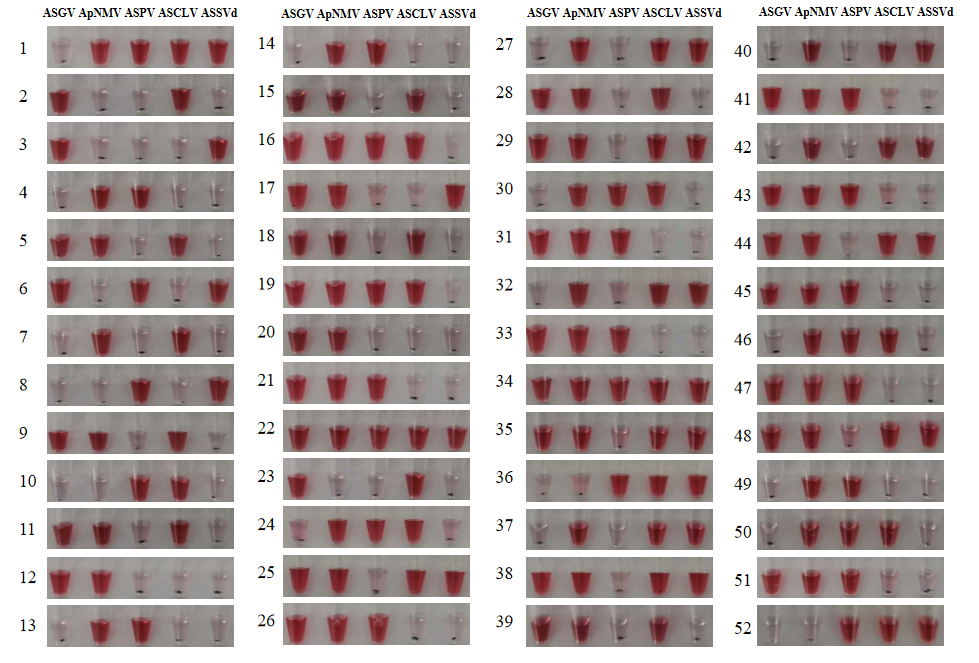

Supplement: Supplementary file 8 — Figure S8 Detection of four RNA viruses and one viroid in 52 apple leaf samples using the RT‐PRA/LbCas12a/AuNP assay. [file PBI-19-394-s008.png]

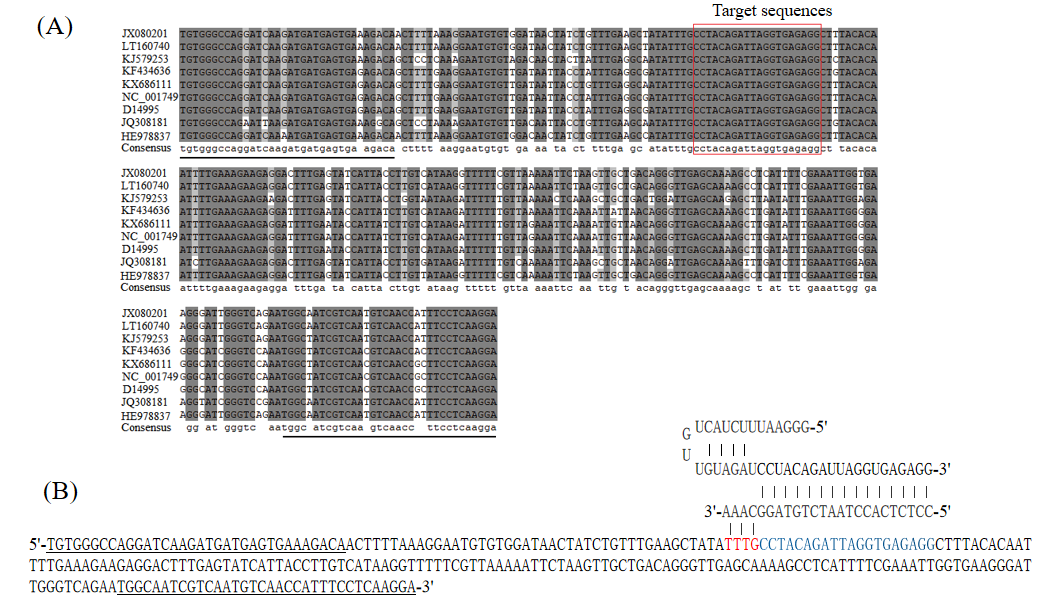

Supplement: Supplementary file 9 — Figure S9 Design of the specific crRNA for ASGV detection. [file PBI-19-394-s009.png]

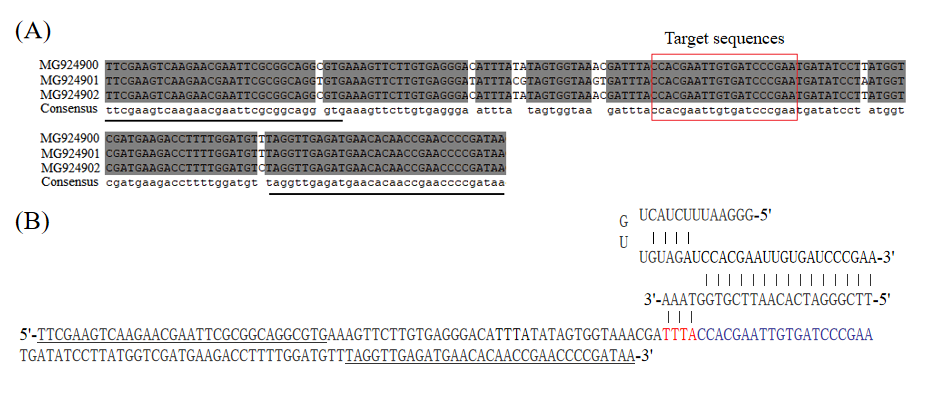

Supplement: Supplementary file 10 — Figure S10 Design of the specific crRNA for ApNMV detection. [file PBI-19-394-s010.png]

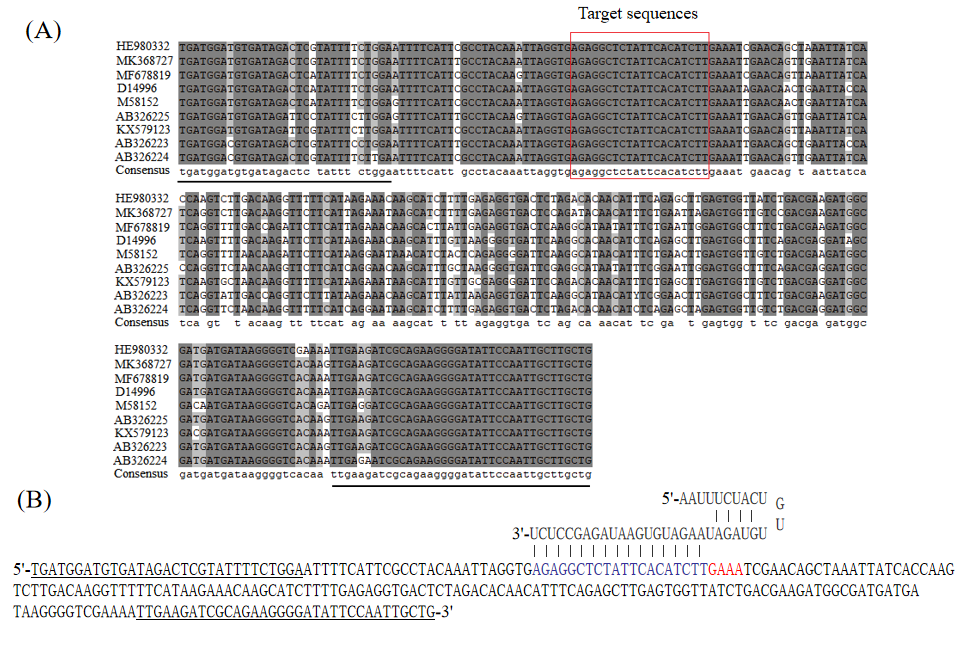

Supplement: Supplementary file 11 — Figure S11 Design of the specific crRNA for ACLSV detection. [file PBI-19-394-s011.png]

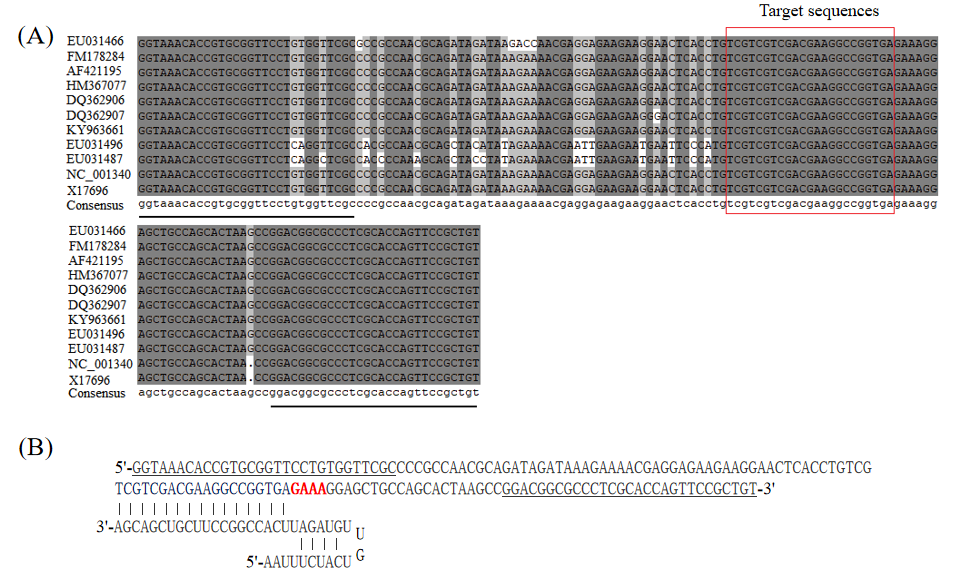

Supplement: Supplementary file 12 — Figure S12 Design of the specific crRNA for ASSVd detection. [file PBI-19-394-s012.png]

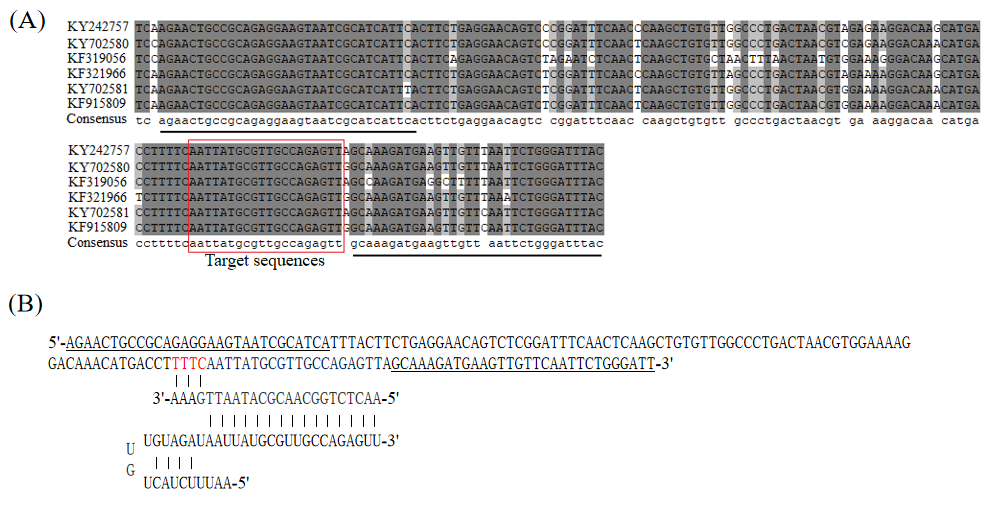

Supplement: Supplementary file 13 — Figure S13 Design of the specific crRNA for ASPV detection. [file PBI-19-394-s013.png]
