# Supplementary material for: Field detection of multiple RNA viruses/viroids in apple using a CRISPR/Cas12a‐based visual assay
Source: Plant Biotechnol J. 2020 Sep 17;19(2):394–405. doi: 10.1111/pbi.13474 (PMC7868969; doi:10.1111/pbi.13474)
Supplement: Supplementary file 14 — Table S1 Comparison of sensitivity among virus/virod detection assays with each of serially diluted RNA standards. Table S2 Comparison between RT‐PCR and RT‐RPA/LbCas12a/AuNP for the detection of four RNA viruses and one viroid in apple leaves collected from different apple varieties from commercial orchards in different regions within Henan Province. Table S3 Number of mixed virus/viroid‐infections in samples collected from commercial fields as detected by the multiplex RT‐RPA/LbCas12a/AuNP visual assay. Table S4 Specific primers for virus/viroid detection by RT‐PCR. Table S5 Targeted sequences for crRNAs and RPA primers Table S6 The primers and TaqMan‐MGB probes used in the one‐step RT‐qPCR assay. Table S7 Oligonucleotides used for preparing transcription templates of crRNAs. Table S8 RT‐RPA primers designed in this study. [file PBI-19-394-s006.doc]

**Table S1. Comparison of sensitivity among virus/virod detection assays with each of serially diluted RNA standards.**

| Targets | One step RT-PCR | One step RT-qPCR | Multiplex RT-RPA/Cas12a/AuNPs | Single-plex RT-RPA/Cas12a/AuNPs |
| --- | --- | --- | --- | --- |
|  | Viral/viroidal copies number per reaction (2.5 ×) | | | |
| ASGV | 10^3^ | 10^2^ | 10^2^ | 10^1^ |
| ASPV | 10^4^ | 10^2^ | 10^2^ | 10^2^ |
| ACLSV | 10^4^ | 10^3^ | 10^3^ | 10^2^ |
| ApNMV | 10^3^ | 10^2^ | 10^3^ | 10^3^ |
| ASSVd | 10^4^ | 10^3^ | 10^3^ | 10^3^ |

**Table S2. Comparison between RT-PCR and multiplex RT-RPA/LbCas12a/AuNP for the detection of four RNA viruses and one viroid in apple leaves collected from different apple varieties from commercial orchards in different regions within Henan Province.**

| Collections | Geographic region | Cultivars | Detection technique | | | | | | | | | |
| --- | --- | --- | --- | --- | --- | --- | --- | --- | --- | --- | --- | --- |
|  |  |  | RT-PCR | | | | | Multiplex RT-RPA/Cas12a/AuNPs | | | | |
|  |  |  | ASGV | ACLSV | ASPV | ApNMV | ASSVd | ASGV | ACLSV | ASPV | ApNMV | ASSVd |
| 1 | Luoning [county, Henan province](http://en.cnki.com.cn/Article_en/CJFDTotal-DIZI200806023.htm) | Golden delicious | - | + | + | + | + | - | + | + | + | + |
| 2 |  |  | + | + | - | - | - | + | + | - | - | - |
| 3 |  |  | + | - | - | - | + | + | - | - | - | + |
| 4 |  |  | - | - | + | + | - | - | - | + | + | - |
| 5 |  |  | + | + | - | + | - | + | + | - | + | - |
| 6 |  |  | + | - | + | - | + | + | - | + | - | + |
| 7 |  | Fuji | - | + | - | + | - | - | + | - | + | - |
| 8 |  |  | - | - | + | - | + | - | - | + | - | + |
| 9 |  |  | + | + | - | + | - | + | + | - | + | - |
| 10 |  |  | - | + | + | - | - | - | + | + | - | - |
| 11 |  |  | + | + | - | + | - | + | + | - | + | - |
| 12 |  |  | + | - | - | + | - | + | - | - | + | - |
| 13 |  |  | - | - | + | + | - | - | - | + | + | - |
| 14 |  |  | - | - | + | + | - | - | - | + | + | - |
| 15 |  |  | + | + | - | + | - | + | + | - | + | - |
| 16 |  | Qinguan | + | + | + | + | - | + | + | + | + | - |
| 17 |  |  | - | - | + | + | + | - | - | + | + | + |
| 18 |  |  | + | + | - | + | - | + | + | - | + | - |
| 19 |  |  | + | + | + | + | - | + | + | + | + | - |
| 20 |  |  | + | - | - | + | - | + | - | - | + | - |
| 21 |  |  | + | - | + | + | - | + | - | + | + | - |
| 22 |  |  | + | + | + | + | + | + | + | + | + | + |
| 23 |  |  | + | + | - | - | - | + | + | - | - | - |
| 24 |  |  | - | + | + | + | - | - | + | + | + | - |
| 25 |  |  | + | + | - | + | + | + | + | - | + | + |
| 26 | Shaanzhou county, [Henan province](http://en.cnki.com.cn/Article_en/CJFDTotal-DIZI200806023.htm) | Fuji | + | - | + | + | - | + | - | + | + | - |
| 27 |  |  | - | + | - | + | + | - | + | - | + | + |
| 28 |  |  | + | + | - | + | - | + | + | - | + | - |
| 29 |  |  | + | + | - | + | + | + | + | - | + | + |
| 30 |  |  | - | + | + | + | - | - | + | + | + | - |
| 31 |  |  | + | - | + | + | - | + | - | + | + | - |
| 32 |  | Starking | + | + | + | - | - | + | + | + | - | - |
| 33 |  |  | + | - | + | + | - | + | - | + | + | - |
| 34 |  |  | + | + | + | + | + | + | + | + | + | + |
| 35 |  |  | + | + | - | + | + | + | + | - | + | + |
| 36 |  |  | - | + | + | - | + | - | + | + | - | + |
| 37 |  |  | - | + | - | + | + | - | + | - | + | + |
| 38 |  |  | + | + | - | + | + | + | + | - | + | + |
| 39 |  |  | + | + | - | + | - | + | + | - | + | - |
| 40 |  |  | + | + | + | - | - | + | + | + | - | - |
| 41 | Lingbao city, [Henan province](http://en.cnki.com.cn/Article_en/CJFDTotal-DIZI200806023.htm) | Fuji | + | - | + | + | - | + | - | + | + | - |
| 42 |  |  | + | + | + | - | - | + | + | + | - | - |
| 43 |  |  | + | - | + | + | - | + | - | + | + | - |
| 44 |  |  | + | + | - | + | + | + | + | - | + | + |
| 45 |  |  | + | - | + | + | - | + | - | + | + | - |
| 46 |  |  | - | + | + | + | - | - | + | + | + | - |
| 47 |  |  | + | - | + | + | - | + | - | + | + | - |
| 48 |  |  | + | + | - | + | + | + | + | - | + | + |
| 49 |  |  | - | - | + | + | - | - | - | + | + | - |
| 50 |  |  | - | + | + | + | - | - | + | + | + | - |
| 51 |  |  | + | - | + | + | - | + | - | + | + | - |
| 52 |  |  | - | + | + | - | + | - | + | + | - | + |
| Positive rate (％) | | | 67.3 | 63.5 | 57.7 | 78.8 | 32.7 | 67.3 | 63.5 | 57.7 | 78.8 | 32.7 |

**Table S3. Number of mixed virus/viroid-infections in samples collected from commercial fields as detected by the multiplex RT-RPA/LbCas12a/AuNP visual assay.**

| Virus combinations | Number of positive samples | Rate |
| --- | --- | --- |
| ASGV+ACLSV | 2 | 3.85% |
| ASGV+ASSVd | 1 | 1.92% |
| ASGV+ApNMV | 2 | 3.85% |
| ASPV+ApNMV | 4 | 7.69% |
| ASPV+ASSVd | 1 | 1.92% |
| ACLSV+ApNMV | 1 | 1.92% |
| ACLSV+ASPV | 1 | 1.92% |
| ASGV+ACLSV+ApNMV | 7 | 13.46% |
| ASGV+ACLSV+ASPV | 3 | 5.77% |
| ASGV+ASPV+ASSVd | 1 | 1.92% |
| ASGV+ASPV+ApNMV | 9 | 17.31% |
| ASPV+ApNMV+ASSVd | 1 | 1.92% |
| ACLSV+ApNMV+ASSVd | 2 | 3.85% |
| ACLSV+ApNMV+ASPV | 4 | 7.69% |
| ACLSV+ASPV+ASSVd | 2 | 3.85% |
| ACLSV+ASPV+ApNMV+ASSVd | 1 | 1.92% |
| ASGV+ASPV+ApNMV+ACLSV | 2 | 3.85% |
| ASGV+ACLSV+ApNMV+ASSVd | 6 | 11.54% |
| ASGV+ASPV+ACLSV+ApNMV+ASSVd | 2 | 3.85% |
| Total | 52 |  |

**Table S4. Specific primers for virus/viroid detection by RT-PCR**

| **Primer name** | **Viruses** | **Sequence（5'-3'）** | **Size（bp）** | **References** |
| --- | --- | --- | --- | --- |
| C-F1 | ACLSV | CAGACCYCTTCATGGAAAGACAG | 725 | H*u et* al (2019) |
| C-R1 |  | GTAGTAAAATATTTAAAAGTCTACAGG |  |  |
| ApNMV-CP+1 | ApNMV | cttgcgtgcaatcgatatgg | 685 | Nod*a et* al (2017) |
| ApNMV-CP-1 |  | tcatctcaacctagacatcc |  |  |
| ASPV-F | ASPV | AAGCATGTCTGGAACCTCAT | 436 | Redesigned |
| ASPV-R |  | AAATCTAGTTAAAACAAAAATAAGC |  |  |
| ASGV-F | ASGV | GGCAGAACTCTTTGAACGAAT | 368 | Redesigned |
| ASGV-R |  | GTATAAAGGCAGGCATGTCAAC |  |  |
| AS1 | ASSVd | CCGGCCTTCGTCGACGACGA | 330 | Pucht*a et a*l (1990) |
| AS3 |  | TGAGAAAGGAGCTGCCAGCAC |  |  |
| ApMV-CP+1 | ApMV | CAAGCGAACCCGAATAAGG | 669 | Nod*a et* al (2017) |
| ApMV-CP-1 |  | ATCACGTACAAATCCCTCAT |  |  |

**Table S5. Targeted sequences for crRNAs and RPA primers**

|  | **Targeted sequence （5'-3'）** | **Genome location** |
| --- | --- | --- |
| 40-nt target dsDNA | NTS-40: TCACGCTCGTCG**TTTG**GTATGGCTTCATTCAGCTCCGGTT  TS-40: AACCGGAGCTGAATGAAGCCATAC**CAAA**CGACGAGCGTGA |  |
| ASGV | NTS:**TGTGGGCCAGGATCAAGATGATGAGTGAAAGACA**ACTTTTAAAGGAATGTGTGGATAACTATCTGTTTGAAGCTATA**TTTG**CCTACAGATTAGGTGAGAGGCTTTACACAATTTTGAAAGAAGAGGACTTTGAGTATCATTACCTTGTCATAAGGTTTTTCGTTAAAAATTCTAAGTTGCTGACAGGGTTGAGCAAAAGCCTCATTTTCGAAATTGGTGAAGGGATTGGGTCAGAA**TGGCAATCGTCAATGTCAACCATTTCCTCAAGGA** | JX080201^a^: 4553 to 4822 |
| ApNMV | NTS:**TTCGAAGTCAAGAACGAATTCGCGGCAGGCGTG**AAAGTTCTTGTGAGGGACATTTATATAGTGGTAAACGA**TTTA**CCACGAATTGTGATCCCGAATGATATCCTTATGGTCGATGAAGACCTTTTGGATGTT**TAGGTTGAGATGAACACAACCGAACCCCGATAA** | MG924900: 1630 to 1794 |
| ASPV | NTS:**AGAACTGCCGCAGAGGAAGTAATCGCATCA**TTTACTTCTGAGGAACAGTCTCGGATTTCAACTCAAGCTGTGTTGGCCCTGACTAACGTGGAAAAGGACAAACATGACC**TTTTC**AATTATGCGTTGCCAGAGTTA**GCAAAGATGAAGTTGTTCAATTCTGGGATT** | KY702581: 72 to 242 |
| ACLSV | TS:**TGATGGATGTGATAGACTCGTATTTTCTGG**AATTTTCATTCGCCTACAAATTAGGTGAGAGGCTCTATTCACATCTT**GAAA**TCGAACAGCTAAATTATCACCAAGTCTTGACAAGGTTTTTCATAAGAAACAAGCATCTTTTGAGAGGTGACTCTAGACACAACATTTCAGAGCTTGAGTGGTTATCTGACGAAGATGGCGATGATGATAAGGGGTCGAAAA**TTGAAGATCGCAGAAGGGGATATTCCAATTGCTG** | HE980332: 5516 to 5771 |
| ASSVd | TS:**GGTAAACACCGTGCGGTTCCTGTGGTTCGC**CCCGCCAACGCAGATAGATAAAGAAAACGAGGAGAAGAAGGAACTCACCTGTCGTCGTCGACGAAGGCCGGTGA**GAAA**GGAGCTGCCAGCACTAAGCC**GGACGGCGCCCTCGCACCAGTTCCGCTGT** | KY963667: 1 to 160 |

a, GenBank accession of representative virus genome; black bolded sequences are the binding domain of RT-RPA primer; underlined region is the target sites of crRNA; PAM sequences are marked in red; NTS, non-target strand; TS, target strand.

**Table S6. The primers and TaqMan-MGB probes used in the one step RT-qPCR assay.**

| **Primers/**  **TaqMan-MGB probes** | **Target** | **Sequence（5'-3'）** | **References** |
| --- | --- | --- | --- |
| 5F | ACLSV | GCCTACAAATTAGGTGAGAGGCTC | Salmo*n et* al (2002) |
| 8R |  | TTCCAATGGATCATGAGGTC |  |
| Mgb26 |  | FAM-ATTCACATCTTGAAATT-MGB |  |
| ApNMV-qf | ApNMV | ACACTCACGCTGGTGGGT | This study |
| ApNMV-qr |  | AAGTCGCTGGTCGCGC |  |
| ApNMV-p |  | FAM-ACCGAGAGCTCGAGCTA-MGB |  |
| qASP-F | ASPV | TGCCTTTTACGCAAAGCATGT | Ioan*na et a*l (2017) |
| qASP-R |  | GTTTGCAGGGGGACTTTGAGT |  |
| ASP-P |  | FAM-TGGAACCTCATGCTGC-MGB |  |
| ASGV-qf | ASGV | GAGTTTGGAAGACGTGCTTCA | Gu*o et* al (2006) |
| ASGV-qr |  | TTGCAGAGAAGAAGGTAAAGCTC |  |
| ASGV-p |  | FAM-CCACCGGGTAGGAGT-MGB |  |
| ASS-F | ASSVd | CCCCTGTTCTCTCACGCTCTT | Malandrak*i et a*l (2015) |
| ASS-R |  | TTTACCGGGAAACACCTATTGTGT |  |
| ASS-P |  | FAM-TGACGCAGCGGCG-MGB |  |

**Table S7. Oligonucleotides used for preparing transcription templates of crRNAs.**

| **Names** | **Targets** | **Sequence（5'-3'）** | **crRNA （5'-3'）** |
| --- | --- | --- | --- |
| T7-crRNA-F |  | GAAATTAATACGACTCACTATAGGG |  |
| T7-TS40-R | TS40 | ggagctgaatgaagccatacATCTACAACAGTAGAAATTCCCTATAGTGAGTCGTATTAATTTC | AAUUUCUACUGUUGUAGAU  GUAUGGCUUCAUUCAGCUCC |
| T7-ASGV-R | ASGV | cctctcacctaatctgtaggATCTACAACAGTAGAAATTCCCTATAGTGAGTCGTATTAATTTC | AAUUUCUACUGUUGUAGAU  CCUACAGAUUAGGUGAGAGG |
| T7-ApNMV-R | ApNMV | ttcgggatcacaattcgtggATCTACAACAGTAGAAATTCCCTATAGTGAGTCGTATTAATTTC | AAUUUCUACUGUUGUAGAU  CCACGAAUUGUGAUCCCGAA |
| T7-ASPV-R | ASPV | aactctggcaacgcataattgATCTACAACAGTAGAAATTCCCTATAGTGAGTCGTATTAATTTC | AAUUUCUACUGUUGUAGAU  CAAUUAUGCGUUGCCAGAGUU |
| T7-ACLSV-R | ACLSV | agaggctctattcacatcttATCTACAACAGTAGAAATTCCCTATAGTGAGTCGTATTAATTTC | AAUUUCUACUGUUGUAGAU  AAGAUGUGAAUAGAGCCUCU |
| T7-ASSVd-R | ASSVd | tcgtcgacgaaggccggtgaATCTACAACAGTAGAAATTCCCTATAGTGAGTCGTATTAATTTC | AAUUUCUACUGUUGUAGAU  UCACCGGCCUUCGUCGACGA |

**Table S8. RT-RPA primers designed in this study**

| **Primer names** | **Targets** | **Sequence（5'-3'）** | **Amplicon length (bp)** |
| --- | --- | --- | --- |
| RPA-SVF | ACLSV | TGATGGATGTGATAGACTCGTATTTTCTGGA | 255 |
| RPA-SVR |  | CAGCAATTGGAATATCCCCTTCTGCGATCTTCAA |  |
| RPA-MVF | ApNMV | TTCGAAGTCAAGAACGAATTCGCGGCAGGCGTG | 165 |
| RPA-MVR |  | TTATCGGGGTTCGGTTGTGTTCATCTCAACCTA |  |
| RPA-PVF | ASPV | AGAACTGCCGCAGAGGAAGTAATCGCATCA | 165 |
| RPA-PVR2 |  | AATCCCAGAATTGAACAACTTCATCTTTGC |  |
| RPA-GVF | ASGV | TGTGGGCCAGGATCAAGATGATGAGTGAAAGACA | 270 |
| RPA-GVR |  | TCCTTGAGGAAATGGTTGACATTGACGATTGCCA |  |
| RPA-VDF | ASSVd | GGTAAACACCGTGCGGTTCCTGTGGTTCGC | 157 |
| RPA-VDR |  | ACAGCGGAACTGGTGCGAGGGCGCCGTCC |  |

**References for tables**

Guo, L.X., Xiang, B.C., Chen, H.Y., Duan, W.J., Chen, H.J. and Zhu, S.F. (2006) Detection of Apple stem grooving virus by real-time fluorescent RT-PCR one step assay. *Acta Phytopathologica Sinica* **36**, 57-61.

Hu, G., Dong, Y., Zhang, Z., Fan, X. and Ren, F. (2019) Elimination of apple necrosis mosaic virus from potted apple plants by thermotherapy combined with shoot-tip grafting. *Scientia Horticulturae* **252**, 310-315.

Ioanna, M., Despoina, B., Ioannis, I., Antonio, O., Christina, V., Nikon, V. and Sek-Man, W. (2017) Simultaneous detection of three pome fruit tree viruses by one-step multiplex quantitative RT-PCR. *Plos one* **12**, e0180877.

Malandraki, I., Varveri, C., Olmos, A. and Vassilakos, N. (2015) One-step multiplex quantitative RT-PCR for the simultaneous detection of viroids and phytoplasmas of pome fruit trees. *Journal of Virological Methods* **213,** 7-12.

Noda, H., Yamagishi, N., Yaegashi, H., Xing, F., Xie, J., Li, S., Zhou, T., Ito, T. and Yoshikawa, N. (2017) Apple necrotic mosaic virus, a novel ilarvirus from mosaic-diseased apple trees in Japan and China. *Journal of General Plant Pathology* **83**, 83-90.

Puchta, H., Luckinger, R., Yang, X.C., Hadidi, A. and Snger, H.L. (1990) Nucleotide sequence and secondary structure of apple scar skin viroid (ASSVd) from China. *Plant Molecular Biology* **14**, 1065-1067.

Salmon, M.A., Vendrame, M., Kummert, J. and Lepoivre, P. (2002) Detection of apple chlorotic leaf spot virus using a 5′ nuclease assay with a fluorescent 3′ minor groove binder-DNA probe. *Journal of Virological Methods* **104**, 99-106.
